# Impact of fatigue in surgeons on performance and patient outcome: systematic review

**DOI:** 10.1093/bjs/znad397

**Published:** 2023-12-14

**Authors:** Iris M Reijmerink, Maarten J van der Laan, J K Götz Wietasch, Lotty Hooft, Fokie Cnossen

**Affiliations:** Department of Surgery, University Medical Centre Groningen, University of Groningen, Groningen, The Netherlands; Department of Surgery, University Medical Centre Groningen, University of Groningen, Groningen, The Netherlands; Department of Anaesthesiology, University Medical Centre Groningen, University of Groningen, Groningen, The Netherlands; Cochrane Netherlands, Julius Center for Health Sciences and Primary Care, University Medical Center Utrecht, Utrecht University, Utrecht, The Netherlands; Department of Artificial Intelligence, Bernoulli Institute of Mathematics, Computer Science and Artificial Intelligence, University of Groningen, Groningen, The Netherlands

## Abstract

**Background:**

While fatigue is an inevitable aspect of performing surgical procedures, lack of consensus remains on its effect on surgical performance. The aim of this systematic review was to assess the effect of non-muscular fatigue on surgical outcome.

**Methods:**

MEDLINE and Embase were searched up to 17 January 2023. Studies on students, learning, duty-hour restrictions, muscle fatigue, non-surgical or subjective outcome, the weekend effect, or time of admission were excluded. Studies were categorized based on real-life or simulated surgery. The Cochrane risk-of-bias tool was used to assess RCTs and the Newcastle–Ottawa scale was used to assess cohort studies. Due to heterogeneity among studies, data pooling was not feasible and study findings were synthesized narratively.

**Results:**

From the 7251 studies identified, 134 studies (including 1 684 073 cases) were selected for analysis (110 real-life studies and 24 simulator studies). Of the simulator studies, 46% (11 studies) reported a deterioration in surgical outcome when fatigue was present, using direct measures of fatigue. In contrast, only 35.5% (39 studies) of real-life studies showed a deterioration, observed in only 12.5% of all outcome measures, specifically involving aggregated surgical outcomes.

**Conclusion:**

Almost half of simulator studies, along with one-third of real-life studies, consistently report negative effects of fatigue, highlighting a significant concern. The discrepancy between simulator/real-life studies may be explained by heightened motivation and effort investment in real-life studies. Currently, published fatigue and outcome measures, especially in real-life studies, are insufficient to fully define the impact of fatigue on surgical outcomes due to the absence of direct fatigue measures and crude, post-hoc outcome measures.

## Teaser

The aim of this systematic review was to assess the effect of non-muscular fatigue on surgical outcome. While most simulator studies that reported an effect of fatigue reported a negative effect, in real-life studies, a large range of findings was found, with inconclusive supporting evidence. While this discrepancy could be explained by heightened motivation and effort investment in real-life studies, the current evidence on fatigue and surgical performance is worrisome; current fatigue and outcome measures used, especially in real-life studies, are insufficient, stemming from the absence of direct fatigue measures and crude, post-hoc outcome measures.

## Introduction

Muscular and non-muscular fatigue are an inevitable aspect of performing surgical procedures^1^, with complex procedures and excessive working hours contributing to fatigue^1,2^. The negative effects of fatigue have even been compared to operating a vehicle with a blood alcohol concentration exceeding the legal limit^3^. While in other professions, for example in the aviation industry, fatigue has been incontrovertibly linked to diminished job functioning and an elevated incidence of errors, the effects of non-muscular fatigue, henceforth referred to simply as ‘fatigue’, on surgical performance are still a subject of controversy^4–6^. Gawande *et al*.^7^ demonstrated that 33% of intraoperative errors were attributable to excessive fatigue, yet Ellman *et al*.^8^ failed to demonstrate a discernible impact of fatigue on surgical outcomes. McCormick *et al*.^9^ found that surgeons can be fatigued for approximately 50% of their waking hours, underscoring the importance of evaluating the potential impact of fatigue on performance to ensure high-quality surgical care. The aim of this systematic review was to assess the impact of non-muscular surgeon fatigue on surgical performance, as measured through both real-life surgery and simulated surgery outcomes.

## Methods

The present study was performed in accordance with PRISMA guidelines and was registered in PROSPERO (CRD42022290704).^10^

### Literature search

MEDLINE and Embase were searched up to 17 January 2023. A search strategy was created by one reviewer (I.M.R.) in consultation with a clinical librarian (*Appendix S1*), using Medical Subject Headings terms and additional free entry terms for non-muscular fatigue, the surgical profession, and clinical competence, and then performed. Reference lists of selected articles and related reviews were screened for other relevant articles.

### Inclusion and exclusion criteria

Inclusion and exclusion criteria were established by three study team members (I.M.R., M.J.v.d.L., and F.C.). One reviewer (I.M.R.) applied these criteria, with uncertainties resolved through discussion with the study team.

Included studies examined the impact of non-muscular fatigue in qualified surgeons and surgical residents in real-life surgery or simulated surgical sessions. In real-life studies, surgical outcome was assessed by patient-based outcome measures, such as number of complications. In simulator studies, surgical outcome was assessed by surgeon-based outcome measures, consisting of either cognitive function or psychomotor function outcome measures, assessed by means of simulated tasks.

Studies on students, learning, duty-hour restrictions, muscle fatigue, non-surgical or subjective outcome, the weekend effect, or time of admission were excluded, as were studies with non-English full text or article types other than original articles. Time-related outcome measures were not included in the analysis, as a decrease in time to task completeness could indicate a learning effect, as well as decreased attention to detail^11,12^.

### Data extraction

Data extraction was performed by one reviewer (I.M.R.) and validated by a second reviewer (Neda Ansari (see the Acknowledgements section)) in a random 10% sample of extracted data. Results from both investigators were compared and any discrepancies were resolved. As no major discrepancies were found, no further verification was undertaken.

Extracted data included research design, type and number of participants, surgical skill studied, setting (that is real-life or simulator), surgical outcome measures studied, fatigue indicators used, and main study findings.

### Quality assessment

One reviewer (I.M.R.) assessed the quality of each included study. The Cochrane risk-of-bias tool was used to assess the quality of RCTs. The Newcastle–Ottawa scale was used to assess the quality of cohort studies^13^. ‘Poor-quality’ cohort studies or studies scoring at high risk of bias in any one of the six bias domains were excluded.

### Data synthesis and analysis

Due to the large heterogeneity of outcome measures and study designs, a meta-analysis was not feasible. Instead, a narrative approach was used to summarize findings. Studies were categorized as either ‘real-life studies’ or ‘simulator studies’ to allow for comparison. See the *Supplementary Methods*.

## Results

**Fig. 1:**
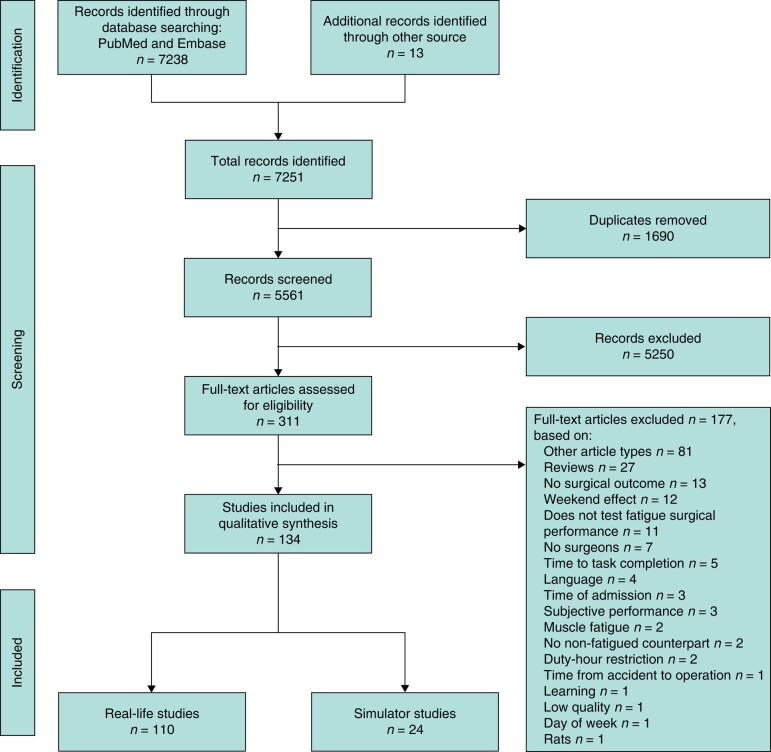
PRISMA flow chart

Out of the 7251 studies identified, a total of 110 real-life studies and 24 simulator studies were included in the analysis (*Fig. 1*). Only one RCT was included in the analysis, scoring a low risk-of-bias for each item^14^.

### Measures of fatigue

Studies used a large variety of measures to establish fatigue in surgeons. See *Table S1* and *Table S2*. In the majority of real-life studies (86 studies), fatigue was assessed based on the time of day of the operation (for example daytime or night-time). Conversely, most simulator studies (17 studies) assessed fatigue in relation to the moment within the on-call shift that the measurement was taken. In contrast to any of the real-life studies, 13 simulator studies utilized validated questionnaires for a more direct measure of fatigue. Additionally, two simulator studies incorporated psychophysiological measurements, such as pupillography, to assess fatigue.

### Surgical outcome in simulator studies

A total of 24 simulator studies, encompassing 460 simulation procedures, were included in the analysis. See *Table S3*. Simulation procedures consisted of either isolated laparoscopic proficiency tasks or simulated surgical operations. Among these studies, 18 studies solely assessed surgical outcome by psychomotor function, whereas 6 studies considered both psychomotor and cognitive functions.

A total of 11 simulator studies (46%) reported negative effects of fatigue on surgical performance. Of these, seven studies reported a decline in one or more psychomotor metrics when fatigue was present, whereas four studies reported a decline in both cognitive and psychomotor metrics, with three of them indicating a more pronounced effect on cognitive performance than on psychomotor performance, as evidenced by significant increases in cognitive errors and impaired performance in tasks requiring cognitive dominance.

Interestingly, one simulator study reported improved cognitive performance when fatigue was present^15^. According to Schlosser *et al*.^15^, this improvement may be rooted in coping strategies and the motivation levels of participants.

Overall, 50% of simulator studies (12 studies) reported no discernible effect on surgical outcome when fatigue was present.

### Surgical outcome in real-life studies

A total of 110 real-life studies, encompassing a total of 1 683 613 surgical procedures, were included in the analysis. See *Table S4*.

Overall, 35.4% of real-life studies (39 studies) reported a deterioration in surgical outcome when fatigue was present, 8.2% of real-life studies (9 studies) reported an improvement in surgical outcome when fatigue was present, 8.2% of real-life studies (9 studies) reported a mixed effect, that is both a deterioration and improvement, when fatigue was present, and 48.2% of real-life studies (53 studies) reported no discernible effect on surgical outcome when fatigue was present.

**Fig. 2:**
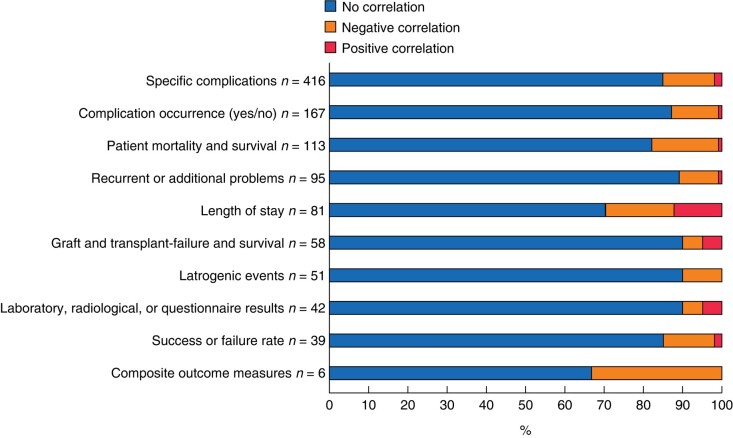
Percentage of outcome measures negatively affected, positively affected, or unaffected by fatigue per category

These studies employed a total of 1068 different patient outcome measures, which could be categorized into 10 distinct groups (*Fig. 2*). When translating the effects to the patient outcome measures, the negative effect of fatigue was present in only 12.5% of all patient outcome measures (134 patient outcome measures), primarily in the category of ‘specific complication mentioned’ (55 patient outcome measures). See *Table S5*.

## Discussion

This systematic review assessed the impact of fatigue on surgical proficiency or outcomes, both in real-life and simulator studies. In simulator studies, fatigue negatively affected surgical performance in almost half of the studies. In contrast, among real-life studies, only one-third of studies showed a deterioration in patient outcomes when fatigue was present. This effect in real-life studies was limited to a subset of the wide-ranging spectrum of patient outcome measures evaluated. However, in the case of real-life studies, a wide range of findings was found and the evidence supporting these effects remains inconclusive.

Almost half of the simulator studies reported a negative effect of fatigue on surgical performance, impairing both cognitive function and psychomotor performance. The majority of identified simulator studies solely focused on psychomotor performance only, despite the known importance of cognitive function performance^16^. Studies reporting both skills showed a more profound decline in cognitive performance^12,17–19^. Many surgical psychomotor skills are highly automatized and maintained during fatigue, for example a surgeon can adequately tie a knot even when fatigued, yet this is not the case for more cognitively demanding tasks^20,21^. While routine operations may be competently completed when a surgeon is fatigued, decision-making in complex cases can never be automated and will always require effort and attention^22^. This aligns with numerous studies in other domains^23,24^.

In real-life studies, a wide range of findings emerged. While some studies assert a negative effect of fatigue, the majority of studies do not report a significant difference in patient outcomes between fatigued and non-fatigued surgeons. Notably, the negative effects observed in the studies are often limited to a very small fraction of the large number and breadth of outcome measures employed.

The negative effects of fatigue, found in half of the simulator studies, seem to diminish in real-life studies, yet a lack of substantive knowledge on fatigue within study designs and flawed methodologies in the real-life studies limit the confidence that this is a true difference. For example, all of the real-life studies and half of the simulator studies lack direct measures of fatigue, such as validated fatigue questionnaires. In most of the real-life studies, the time of day of the operation is used as a proxy for fatigue, which assumes, without validation, that surgeons experience fatigue during night shifts^25^. Furthermore, in real-life surgery, outcomes rely heavily on crude indicators of patient outcome, such as patient mortality or number of readmissions, which are impacted by multiple other factors and are typically analysed at an aggregated level rather than at the individual surgeon level. In contrast, simulator studies directly examine a surgeon’s cognitive or psychomotor performance. By incorporating more specific behavioural outcome measures within otherwise controlled environments, simulator studies have the potential to detect more subtle differences between fatigued and non-fatigued surgeons. Finally, while some real-life studies report a significant association between surgeon fatigue and patient outcome, it is not possible to establish a direct causal relationship based solely on these findings given other potential variables impacting patient outcome, for example differences between and within surgical teams and in perioperative care. All such differences can influence outcomes, leading to different results, even among patients with similar baseline conditions, regardless of a surgeon’s state of fatigue. Consequently, while the aggregated measures used in these studies may provide a comprehensive overview of the collective surgical team’s performance and its broader context, they fail to accurately represent an individual surgeon’s contributions.

Fatigue emerges from prolonged engagement in a demanding task within a context of limited control. In high-stake situations (that is in real-life studies), individuals naturally strive to uphold performance at a satisfactory level by channeling increased effort^26^. High intrinsic motivation can counteract the fatigue-induced impact on performance^15,27–30^. However, while sustained high effort might temporarily mitigate the effects of fatigue, it can culminate in the experience of strain, a state associated with burnout^31^. Thus, surgical performance may be protected, but at a cost to the surgeon^32^.

Given the current methodological shortcomings, accurately assessing the true impact of fatigue on surgeon performance remains challenging. The findings of the present study support the need for future research to incorporate kinematic measures by means of instrument position trackers and accelerometers into real-life surgery^33,34^. Additionally, integrating validated fatigue detection methods is imperative. Approaches, such as electrocardiography and eye metrics, which have already demonstrated effectiveness in detecting fatigue within the aviation industry, could prove valuable^35,36^. By combining these kinematic and fatigue measurement approaches, more insight may be gained into the real effects of fatigue on surgical performance and also patient outcome. Furthermore, future studies should embrace a broader lens, encompassing team performance rather than focusing solely on the individual surgeon. Such an approach will enrich the understanding of the intricate interplay between various elements within the surgical environment.

This study has some inherent limitations. First, there was large heterogeneity in fatigue measures amongst the studies involved, as well as statistical analyses applied, complicating comparisons between studies. Furthermore, as almost all studies focusing on after-hours or sleep deprivation mentioned fatigue, it was assumed that the goal of these studies was to examine the effects of fatigue.

To conclude, almost half of the simulator studies and one-third of the real-life studies reported negative effects of fatigue, highlighting a significant patient safety issue. While in real-life studies, a difference in motivation might protect task performance, the current fatigue and outcome measures used, especially in real-life studies, are insufficient to accurately determine whether fatigue truly impacts surgical outcomes. Further research should include more direct measures of a surgeon’s performance in real-life studies, measures of the effects on the whole surgical team, and a core clinical outcome set for surgical outcomes.

## Supplementary Material

znad397_Supplementary_Data

## Data Availability

The data that support the findings of this study are derived from public domain sources and are openly available within MEDLINE and Embase.
