# Supplementary material for: Impact of fatigue in surgeons on performance and patient outcome: systematic review
Source: Br J Surg. 2023 Dec 14;111(1):znad397. doi: 10.1093/bjs/znad397 (PMC10771255; doi:10.1093/bjs/znad397)
Supplement: znad397_Supplementary_Data [file znad397_supplementary_data.docx]

**The impact of fatigue in surgeons on performance and patient outcome; systematic review**

Authors: Iris M. Reijmerink (1), Maarten J. van der Laan (1), J.K. Götz Wietasch (2), Lotty Hooft (3), Fokie Cnossen (4)

1. Department of Surgery, University Medical Centre Groningen, Groningen, The Netherlands
2. Department of Anesthesiology, University Medical Centre Groningen, Groningen, The Netherlands
3. Cochrane Netherlands, Julius Center for Health Sciences and Primary Care, University Medical Center Utrecht, Utrecht University, Utrecht, The Netherlands
4. Department of Artificial Intelligence, Bernoulli Institute of Mathematics, Computer Science and Artificial Intelligence, University of Groningen, Groningen, the Netherlands

Corresponding author: Iris M Reijmerink
Department of Surgery (Division of Vascular Surgery), University Medical Center Groningen, University of Groningen, Groningen, The Netherlands.
Hanzeplein 1, 9713 GZ Groningen, The Netherlands.

E-mail address: [i.m.reijmerink@umcg.nl](mailto:i.m.reijmerink@umcg.nl)

ORCID ID: 0000-0002-2095-2215

**Supplementary Materials - Index**

| **Supplementary Methods** | *Page 2* |
| --- | --- |
| **Supplementary Appendixes** |  |
| Appendix 1 – Search strategy | *Page 5* |
| **Supplementary Figures and Tables** |  |
| Supplementary Table 1: Measures used to assess fatigue in real-life studies. | *Page 6* |
| Supplementary Table 2: Measures used to assess fatigue in simulator studies. | *Page 14* |
| Supplementary Table 3: Outcome simulator studies between fatigued and non-fatigued surgeons. | *Page 18* |
| Supplementary Table 4: Outcome real-life studies between fatigued and non-fatigued surgeons. | *Page 24* |
| Supplementary Table 5: Outcome measures included in real-life studies | *Page 49* |
| **References** | *Page 66* |
|  |  |

**Supplementary Methods**

The current study was performed in accordance with the Preferred Reporting Items for Systematic Reviews and Meta-Analyses (PRISMA) guidelines and was registered on PROSPERO, registration number CRD42022290704. As this study did not utilize individual patient data, no ethical approval was required for this study.

**Literature search**

Medline and EMBASE database were searched up to January 17^th^, 2023. A search strategy was created by the investigator (IMR) in consultation with a clinical librarian. (Appendix 1) Medical Subject Headings terms and additional free entry terms for non-muscular fatigue, the surgical profession, and clinical competence were used. There were no restrictions regarding the year of publication. Prior to data analysis, both exact and close duplicates were removed after manual inspection using Refworks. Reference lists of selected articles were screened for other relevant articles.

**Inclusion and exclusion criteria**

A predetermined set of inclusion and exclusion criteria was used to select potentially eligible studies for full text analysis. Studies that were included described the effect of non-muscular fatigue of surgeons and surgical residents on either real-life surgery or simulated surgical sessions. In the studies focusing on real-life surgery, surgical outcome was assessed based on patient-based outcome measures, such as number of blood transfusions, complications, and number of days in hospital. In the studies focusing on simulated surgery, surgical outcome was assessed based of surgeon-based outcome measures. Surgeon-based outcomes consisted of either cognitive-function (CF) outcome measures, i.e., error explicitly stated to be of cognitive nature, or psychomotor-function (PF) outcome measures, e.g., instrument movement smoothness, both assessed by means of simulated tasks. Cognitive- or psychomotor function assessed by other means than simulated tasks, such as factual recall tasks, were not included in the analysis.

After the primary analysis to select eligible studies for full text analysis, we decided that time might not be a reliable measure of outcome for surgical performance. It is questionable whether a decrease in task completion time suggests an increase in performance, as it could indicate a learning effect or a smooth intraoperative process, as well as less attention to detail.^10,11^ Time-related outcomes such as time to task completeness and operation duration were therefore not included in the final analysis after all. Regarding participants, studies including non-surgical professions or medical students were excluded from the analysis. Regarding outcome, studies based on non-surgical outcomes, subjective outcomes (e.g., surveys or expert opinions), surgery performed on animals, or studies focused on learning were excluded from the analysis. Regarding fatigue measures, studies based on direct measures of fatigue, post-call, sleep deprivation and night-time operations were included. Studies based on muscle fatigue, the weekend-effect, or time-of-admission instead of time-of-operation were excluded, as were studies who did not include a non-fatigued counterpart. As well as time-related outcome measures, after the primary analysis to select eligible studies for full-text analysis, we decided to also exclude studies on duty-hour restriction, as it is questionable whether a change in restriction represents fatigue. Finally, studies with non-English full text and article types other than original articles, such as letters to the editor, comments, and conference abstract, were excluded. Reviews were not included in the analysis, but review reference lists were screened for potentially relevant additional publications. The selection criteria were created by three members of the study team (IMR, MJL, FC), which were then applied by one researcher (IMR). Uncertainties in inclusion screening were resolved by discussion with the study team.

**Data extraction**

Data from all eligible studies were extracted using standardized data extraction tables developed prior the start of the study in Microsoft Office Excel (V.2016l Microsoft, Redmond, Washington, USA). One reviewer (IMR) independently extracted data from the eligible studies. Data extraction was verified by a second reviewer (NA) using a random 10% sample. Results from both investigators were compared for discrepancies. Minor discrepancies were discussed and solved between the two investigators (IMR, NA). As no major errors or discrepancies were recognized, no further verification was undertaken.

The data extracted from the studies included research design, type and number of participants, surgical skill studied, setting (i.e., real-life, or simulator), surgical outcome measures studied, fatigue indicators used, and main findings of the study. Studies missing information about any of these data extractions were excluded from the analysis.

**Quality assessment**

The quality of each included study was critically assessed by one reviewer (IMR). The Cochrane Risk of Bias Tool was used to assess randomized controlled trials (RCTs). The Newcastle-Ottawa Scale was used to appraise the methodological quality and certainty assessment of cohort studies included, by providing a score to three factors: (1) selection, including the representativeness of the exposed cohort, selection of the non-exposed cohort, ascertainment of exposure, and demonstration that outcome of interest was not present at start of the study; (2) comparability, namely the comparability of cohorts and the controlling of confounders factors; and (3) outcome, including the assessment of outcome, whether follow-up was long enough for outcomes to occur, and the adequacy of follow-up of cohorts.^12^ “Poor quality” studies – studies with 0-1 star for selection, 0 stars for comparability or 0-1 start for outcome, as well as studies scoring a high risk on one of the six bias domains, were excluded.

**Data synthesis and analysis**

Due to the large heterogeneity of surgical outcome measures, fatigue indicators and research design, performing a meta-analysis was not possible. To synthesize the study findings, results were therefore presented by a narrative approach. A subgroup analysis was performed: studies were categorized as either “real-life studies” or “simulator studies’, depending on whether surgical outcomes were assessed after a real-life surgical intervention or results were derived from simulated procedures. This categorization draws an analogy to in-vivo/in-vitro research, allowing for examination in the differences in severity and consequence in real-life studies and controllability of outcome parameters in simulator studies.

Results from both types of studies were compared. Only outcome measures with available summary statistics were included in the analysis, while any outcomes without summary statistics (e.g., missing p-value) were excluded. The findings were presented in a tabular form. Results were summarized as “no significant effect” under two conditions: firstly, when no effect was observed in the initial univariate analysis; second, when an effect was detected in the univariate analysis but dissipated in any subsequent multivariate analysis.

**Supplementary Appendixes**

**Appendix 1: Search strategy**

***Pubmed search strategy:***

("Fatigue"[Mesh] OR fatigue[tiab] OR "Sleep Deprivation"[Mesh] OR sleep depriv*[tiab] OR sleep insufficien*[tiab] OR exhaustion[tiab] OR tired*[tiab] OR night* work[tiab] OR nighttime[tiab] OR 80-hour work*[tiab] OR duty hour restriction*[tiab] OR time of day[tiab] OR time-of-day[tiab] OR after-hour* surgery[tiab])

AND

(("General Surgery"[Mesh] OR "Surgical Procedures, Operative"[Mesh:NoExp] OR surgery[tiab] OR surgeon[tiab] OR surgical[tiab])

AND ("Psychomotor Performance"[Mesh] OR "Time Factors"[Mesh] OR "Clinical Competence"[Mesh] OR psychomotor performance[tiab] OR clinical competence[tiab] OR "Surgeons"[Mesh] OR surgeon*[tiab] OR operation time[tiab] OR operating time[tiab] OR long operation*[tiab] OR longer operation*[tiab] OR duration of surg*[tiab] OR duration of operat*[tiab] OR duration of proced*[tiab] OR during surg*[tiab] OR surgical skill*[tiab] OR surgical pract*[tiab] OR surgical performan*[tiab] OR surgical resident*[tiab] OR surgical outcome[tiab] OR operative outcome*[tiab] OR outcome of proced*[tiab] OR adverse outcome*[tiab]))

*2589 hits 17-01-2023*

***EMBASE search strategy:***

('fatigue'/exp OR 'sleep deprivation'/exp OR fatigue:ti,ab OR 'sleep depriv*':ti,ab OR 'sleep insufficien*':ti,ab OR exhaustion:ti,ab OR tired*:ti,ab OR 'night* work':ti,ab OR nighttime:ti,ab OR '80-hour work*':ti,ab OR 'duty hour restriction*':ti,ab OR 'time of day':ti,ab OR 'time-of-day':ti,ab OR 'after-hour* surgery':ti,ab) AND ('surgery'/exp OR surgery:ti,ab OR surgeon:ti,ab OR surgical:ti,ab) AND ('psychomotor performance'/exp OR 'time factor'/exp OR 'clinical competence'/exp OR 'surgeon'/exp OR 'psychomotor performance':ti,ab OR 'clinical competence':ti,ab OR surgeon*:ti,ab OR 'operation time':ti,ab OR 'operating time':ti,ab OR 'long operation*':ti,ab OR 'longer operation*':ti,ab OR 'duration of surg*':ti,ab OR 'duration of operat*':ti,ab OR 'duration of proced*':ti,ab OR 'during surg*':ti,ab OR 'surgical skill*':ti,ab OR 'surgical pract*':ti,ab OR 'surgical performan*':ti,ab OR 'surgical resident*':ti,ab OR 'surgical outcome':ti,ab OR 'operative outcome*':ti,ab OR 'outcome of proced*':ti,ab OR 'adverse outcome*':ti,ab)

*4649 hits 17-01-2023*

**Supplementary Figures and Tables**

**Supplementary Table 1: Measures used to assess fatigue in real-life studies.**

| **Source** | **Operationalization of fatigue** | | **Other measures of fatigue ^(A)^** |
| --- | --- | --- | --- |
|  |  |  | **Non-validated** |
| A-lai, 2022.^36^ | Time of day of operation | *Early start (8:00/09:00 – 16:00) or late start (16:00 – 19:00).* |  |
| Ahlsson, 2019.^37^ | Time of day of operation | *Daytime (08:00 – 20:00) or nighttime (20:00 – 08:00).* |  |
| Alnajashi, 2020.^38^ | Time of day of operation | *Morning (08:00 – 15:59), evening (16:00 – 23:59), or night (00:00 – 07:59).* |  |
| Araujo, 2014.^39^ | Time of day of operation | *Morning vs. afternoon (before or after 12:00), as well as at 07:00 - 11:00, 11:01 - 15:00, or 15:01 and later.* |  |
| Arnaoutakis, 2020.^40^ | Time of day of operation | *Daytime (08:00 – 17:00) or nighttime (17:00 – 08:00).* |  |
| Assali, 2006.^41^ | Time of day of operation | *Daytime (08:00 –18:00) or nighttime (18:00 – 08:00).* |  |
| Axtell, 2020.^42^ | Time of day of operation | *Early (07:00 – 15:00) or Late (15:00 and later).* |  |
| Aydoğmuş, 2017.^43^ | Time of day of operation | *Daytime (08:00 – 17:00) or nighttime (17:00 – 07:59).* |  |
| Bagrodia, 2012.^44^ | Work hours | *First or second operation of the day.* |  |
| Bailit, 2006.^45^ | Time of day of operation | *Morning. (07:00 – 15:00), evening (15:00 – 23:00) or night (23:00 – 07:00).* |  |
| Barinaga, 2017.^46^ | Time of day of operation | *Daytime (07:00 – 17:00) or nighttime (17:00 – 07:00).* |  |
| Becker, 2019.^47^ | Time of day of operation | *Daytime (08:00 – 18:00) or nighttime (18:00 – 08:00).* |  |
| Bekelis, 2018.^48^ | Post call vs. non-post call | *Whether surgeon operated the night before surgery.* |  |
| Bianco, 2021.^49^ | Time of day of operation | *Start times from 07:00 – 11:00 or 15:00 – 23:00.* |  |
| de Boer, 2018.^50^ | Time of day of operation | *Daytime (08:00 – 17:00) or nighttime (17:00 – 08:00).* |  |
| Boscà, 2019.^51^ | Work hours; Time of day of operation | *>12 or <12 hours passed since previous procedure; Daytime (08:00 – 22:00) or nighttime (22:00 – 08:00).* |  |
| Canal, 2020.^52^ | Time of day of operation | *Morning (07:00 – 12:59), afternoon (13:00 – 18:59), evening (19:00 – 23:59), or night (00:00 – 06:59).* |  |
| Capello, 2008.^53^ | Work hours | *First, second or third operation of the day.* |  |
| Chacko, 2011.^54^ | Time of day of operation | *Daytime (07:00 – 17:59) or nighttime (18:00 – 05:59).* |  |
| Chan, 2018.^55^ | Time of day of operation | *Daytime (08:00 – 16:59), out of hours before midnight (17:00 – 23:59) or after midnight (00:00 – 07:59).* |  |
| Chang, 2008.^56^ | Time of day of operation | *Start time at 8.30 –10.29; 11.30 –13.29 or 15.30 –17.29 hours.* |  |
| Chen, 2020.^57^ | Time of day of operation | *Daytime (08:00 – 18:00) or nighttime (18:00 and later).* |  |
| Chiu, 2019.^58^ | Time of day of operation | *Daytime (08:00 – 16:59) or nighttime (17:00 – 06:00).* |  |
| Chu, 2011.^59^ | Sleep deprived (SD) vs. non-sleep deprived (NSD) | *SD = Surgeon slept 0 – 3 or 3 – 6 hours.*  *NSD = surgeon slept >6 hours.* |  |
| Cook, 1997.^60^ | Time of day of operation | *Day (08:00 – 17:00), evening (17:01 – 22:00) or night (22:01 – 07:59).* |  |
| Coumbe, 2011.^61^ | Time of day of operation | *Before or after 16:00.* |  |
| Dalton, 2016.^62^ | Time of day of operation | *Daytime (06:00 - 23:00) or nighttime (23:00 – 06:00).* |  |
| Ellman, 2004.^63^ | Post call vs. non-post call | *Post call = surgeon started case between 22:00 – 05:00, or ended a case between 23:00 – 07:30, and performed a subsequent case in the next 24 hours.*  *Non-post call = all other cases.* |  |
| Ellman, 2005.^8^ | Post call vs. non-post call | *Post call = surgeon started case between 22:00 – 05:00, or ended a case between 23:00 – 07:30, and performed a subsequent case in the next 24 hours.*  *Non-post call = all other cases.* |  |
| Eskesen, 2018.^64^ | Time of day of operation | *Day (06:00 – 14:00), evening (14:00 – 22:00) or night (22:00 – 06:00).* |  |
| Fechner, 2008.^65^ | Time of day of operation | *Daytime (08:00 – 20:00) or nighttime (20:00 – 08:00).* |  |
| Fernandes, 2016.^66^ | Time of day of operation | *Daytime (08:00 – 20:59) or nighttime (21:00 – 07:59).* | Hours worked in week preceding shift period; Hours slept preceding 24 hours; Fatigue level during nighttime (urgency shift). |
| Gabriel, 2018.^67^ | Time of day of operation | *Day (07:00 – 17:00), evening (17:01 – 00:00) or night (00:01 – 06:59).* |  |
| Gasser, 2020.^68^ | Time of day of operation | *Daytime (05:00 – 19:00) or nighttime (19:01 – 04:59).* |  |
| George, 2011.^69^ | Time of day of operation | *Daytime (07:0 – 19:00) or nighttime (19:00 – 07:00).* |  |
| Goertz, 2021.^70^ | Time of day of operation | *Daytime (08:00–17:59) or nighttime (18:00–07:59).* |  |
| Govindarajan, 2015.^71^ | Post call vs. Non-post call | *Physician had worked clinically in the preceding over-night hours (midnight – 07:00) or not.* |  |
| Guidry, 2016.^72^ | Time of day of operation | *Morning (06:00 – 11:59), afternoon (12:00 – 17:59), evening (18:00 – 23:59) or night (00:00 – 05:59).* |  |
| Guo, 2019.^73^ | Time of day of operation | *Daytime (08:00 – 20:00) or nighttime (20:0 – 08:00).* |  |
| Halldorson, 2009.^74^ | Work hours | *<24h, 24-48h, 48-72h, 72-96h or >96h between the first and consecutive operation.* |  |
| Halliday, 2019.^75^ | Time of day of operation | *Daytime (07:00 – 19:00) or nighttime (19:00 – 07:00).* |  |
| Halvachizadeh, 2019.^76^ | Time of day of operation | *Morning (07:00 – 12:59), afternoon (13:00 – 18:59), evening (19:00 – 23:59) or night (00:00 – 06:59).* |  |
| Haynes, 1995.^77^ | Post call vs non-post call | *Post call = on call the day before the operation for 24 hours without leaving the hospital.*  *Non-post call = all other cases.* | Sleep survey (current rotation, hours slept during last night on in-house call; hours usually slept while on in-house call; whether sleep lacked with performance). |
| Heller, 2017.^78^ | Time of day of operation | *Daytime (06:00 – 16:00) or nighttime (16:01 – 05:59).* |  |
| Hsu, 2015.^79^ | Time of day of operation | *Morning (06:00 – 12:00), afternoon (12:00 – 17:00) or evening / night (17:00 – 06:00).* |  |
| Ishiyama, 2019.^80^ | Work hours | *Morning (<13:00) or consecutively in the afternoon (>13:00).* |  |
| Karagoz, 2022.^81^ | Time of day of operation | *Daytime (08:00 – 17:00) or after-hours (17:01 – 07:59).* |  |
| Kelz, 2008.^82^ | Time of day of operation | *At 07;00 - 15:59, 16:00 - 17:59, 18:00 - 22:59 or 23:00 - 06:59.* |  |
| Kelz, 2009.^83^ | Time of day of operation | *At 07:30- 09:30 (reference interval), 09:30 - 11:30, 11:30 - 13:30, 13:30 - 15:30, 15:30 - 17:30, 17:30 - 19:30, 19:30 - 21:30 or 21:30 - 07:30.* |  |
| Kienzl-Wagner, 2013.^84^ | Time of day of operation | *Daytime (08:00 – 20:00) or nighttime (20:00 – 08:00).* |  |
| Kim, 2022.^85^ | Time of day of operation | *At 08:00 – 18:00, 18:00 – 20:00 or 20:00 – 08:00.* |  |
| Koltka, 2018.^86^ | Time of day of operation | *Day (08:01 – 17:00), early after-hours (17:01 – 23:00) or night (23:01 – 08:00).* |  |
| Kork, 2018.^87^ | Time of day of operation | *Hour of day.* |  |
| Larsen, 2017.^88^ | Time of day of operation | *Daytime (07:00 – 23:00) or nighttime (23:00 – 07:00).* |  |
| Lee, 2013.^89^ | Time of day of operation | *Daytime (07:00 – 16:00) or after-hours (16:00 – 07:00).* |  |
| Li, 2018.^90^ | Work hours | *First, second, third or fourth/later operation of the day.* |  |
| Lim, 2015.^91^ | Time of day of operation | *Daytime (07:30 – 17:00) or after-hours.* |  |
| Lonze, 2010.^92^ | Time of day of operation | *Daytime (03:00 – 15:00) or nighttime (15:00 – 03:00).* |  |
| Lu, 2017.^93^ | Time of day of operation; Work hours | *Morning (08:00 – 13:00) or afternoon (13:00 – 18:00); Surgeon performing afternoon surgery (start-time 13:00 – 18:00) had performed other surgeries earlier that day or not.* |  |
| Lu, 2020.^94^ | Time of day of operation | *Morning (08:00 – 13:00) or afternoon (13:00 – 18:00).* |  |
| Mehra, 2020.^95^ | Time of day of operation | *Daytime (08:00 – 20:00) or nighttime (20:00 – 08:00).* | Custom-made questionnaire on urgent renal transplant and fatigue (whether nighttime is period of lesser ability; median no. hours sleep (+when transplant start between 18:00 – 06:00); general fatigue level at nighttimel whether emergency renal transplant could be postponed to following day). |
| Miyahara, 2021.^96^ | Post call vs. non-post call | *On nighttime duty (from 17:00 – 08:30)*  *the night before surgery or not.* |  |
| Mönttinen, 2021.^97^ | Time of day of operation | *Daytime (08:00 – 22:00) or nighttime (22:00 – 08:00).* |  |
| Narayan, 2020.^98^ | Time of day of operation | *Daytime (07:00 – 19:00) or nighttime (19:00 – 07:00).* |  |
| Nasseri, 2022.^99^ | Time of day of operation | *Morning (before 12:00) or afternoon (after 12:00).* |  |
| Ndegbu, 2019.^100^ | Time of day of operation | *Daytime (08:00 – 19:59) or nighttime (20:00 – 07:59).* |  |
| Okkaoglu, 2021.^101^ | Time of day of operation | *Working hours (08:00 -17:00), night (17:00 – 0:00) or late night (0:00 – 8:00).* |  |
| Orman, 2012.^102^ | Time of day of operation | *Daytime (07:00 – 19:00) or nighttime (19:00 – 07:00).* |  |
| Özdemir-van Brunschot, 2016.^103^ | Time of day of operation | *Daytime (08:00 – 20:00) or nighttime (20:00 – 08:00).* |  |
| Patel, 2014.^104^ | Time of day of operation | *Daytime (07:00 – 18:59) or nighttime (19:00 – 06:59, and 19:00 – 22:59, 23:00 – 02:59, and 03:00 – 06:59).* |  |
| Patel, 2018.^105^ | Time of day of operation | *Day (08:00 – 17:00), evening (17:00 – 00:00) or night (00:00 – 08:00).* |  |
| Patella, 2017.^106^ | Time of day of operation | *Morning (before 12:00) or afternoon (after 12:00).* |  |
| Peled, 2011.^107^ | Time of day of operation | *Morning (07:00 – 15:00), evening (15:00 – 23:00), or night (23:00 – 07:00).* |  |
| Peskun, 2012.^108^ | Time of day of operation | *Hour of day.* |  |
| Phatak, 2014.^109^ | Time of day of operation | *Daytime (07:00 – 23:00) or nighttime (23:00 – 07:00).* |  |
| Qiu, 2018.^110^ | Time of day of operation | *Daytime (08:00 – 17:00) or nighttime (17:00 – 08:00).* |  |
| Rashid, 2013.^111^ | Time of day of operation | *Daytime (07:00 – 17:00) or nighttime (17:00 – 07:00).* |  |
| Ricci, 2009.^112^ | Time of day of operation | *Daytime (06:00 – 16:00) or after-hours (16:00 – 06:00).* |  |
| Rogers, 2020.^113^ | Time of day of operation | *Daytime (07:30 – 16:59) or nighttime (17:00 – 7:29).* |  |
| Rothschild, 2009.^114^ | Post call vs. Non-post call; Sleep deprived (SD) vs. non-sleep deprived (NSD) | *Whether surgeon operated night before; Sleep opportunity 0 – 6h or >6h (time between end last overnight procedure and the beginning first post-nighttime procedure); Work duration 0 – 12h or >12h (time from the start first overnight procedure to end post-nighttime procedure).* |  |
| Schieman, 2008.^115^ | Post call vs. non-post call | *Post call = surgeon billed for clinical work after 22:00 night before operation; non-post call = all other cases.* |  |
| Schuster, 2018.^116^ | Sleep deprived (SD) vs. Non-sleep deprived (NSD) | *SD = case started after midnight and surgeon had no sleep in prior 18 hours. NSD = case started at or before 20:00 or the surgeon slept for at least 3 of the 6 hours preceding the case.* |  |
| Seklehner, 2016.^117^ | Work hours | *<6 hours, 6-12 hours or >12 hours worked before performing surgery.* |  |
| Seow, 2004.^118^ | Time of day of operation | *Day (07:30 – 17:59), evening (18:00 – 23:59) or night (00:00 – 07:29).* |  |
| Sessler, 2011.^119^ | Time of day of operation | *Hour workday (06:00 – 19:00).* |  |
| Shah, 2022.^120^ | Time of day of operation | *Day-time (07:00 – 13:00 and/or 13:00 – 17:00) or nighttime (19:00 – 01:00 and/or 01:00 – 07:00).* |  |
| Sharpe, 2013.^121^ | Post call vs. non-post call | *Post call = operations performed day after surgeon had overnight trauma shift (from 17:00 – 07:00). Non-post call = all other cases.* |  |
| Shaw, 2012.^122^ | Time of day of operation | *Daytime (06:00 – 18:00) or nighttime (18:00 – 06:00), as well as during what 3-hour block.* |  |
| Siada, 2017.^123^ | Time of day of operation | *Daytime (07:00 – 17:00) or nighttime (17:00 – 07:00).* |  |
| Sugünes, 2019.^124^ | Time of day of operation | *Daytime (08:00 – 20:00) or nighttime (20:00 – 08:00).* |  |
| Sun, 2022.^125^ | Work hours | *Whether surgeon operated night before (23:00 – 07:00), and length of time worked night before (none, >0 to <2, 2 to <4, 4 to <6, or 6-8).* |  |
| Switzer, 2013.^126^ | Time of day of operation | *Daytime (07:00 – 15:59) or nighttime (16:00 – 06:59).* |  |
| Tan, 2009.^127^ | Time of day of operation | *Hour of day.* |  |
| Tessler, 2018.^128^ | Time of day of operation | *Day (07:30 – 15:30), evening (15:30 – 23:30) or night (23:30 – 07:30).* |  |
| Thomas, 2012.^129^ | Workload | *Daily surgeon workload (number of rooms used, operations performed and total operative time that day).* |  |
| Treacy, 2022.^130^ | Time of day of operation | *Daytime (08:00 – 18:30) or nighttime (18:30 – 08:00).* |  |
| Tu, 2021.^131^ | Time of day of operation | *Before or after 17:00.* |  |
| Tu, 2023.^132^ | Time of day of operation | *Daytime (08:00 – 17:29) or after-hours (17:30 – 23:59).* |  |
| Turrentine, 2010.^133^ | Time of day of operation | *Daytime (07:00 – 18:59) or nighttime (19:00 – 06:59).* |  |
| Vimalesvaran, 2013.^134^ | Time of day of operation | *Routine hours (09:00 – 18:00) or out-of-hours (18:01- 08:59).* |  |
| Vinden, 2013.^135^ | Post call vs. non-post call | *Operated night before or not.* |  |
| Wan, 2021.^136^ | Work hours | *<5 hours or 5-10 hours worked prior to performing surgery.* |  |
| Wang, 2020.^137^ | Time of day of operation | *Morning or afternoon (before or after 11:23 (median start time) or 13:00).* |  |
| Wendling-Keim, 2022.^138^ | Time of day of operation | *Hour of day.* |  |
| Wu, 2014.^139^ | Time of day of operation | *Daytime (07:00 – 19:00) or nighttime (19:00 – 07:00).* |  |
| Yaghoubian,  2010.^140^ | Work hours | *Surgery completed during daytime (06:00 – 22:00) or nighttime (22:00 – 06:00) after working day.* |  |
| Yang, 2021.^141^ | Time of day of operation | *Daytime (05:00 – 18:00) or nighttime (180 – 05:00).* |  |
| Yount, 2015.^142^ | Time of day of operation | *Before or after 15:00.* |  |
| Van Zaane, 2015.^143^ | Time of day of operation | *Day (08:01 - 17:00), evening (17:01 - 24:00) or night (0:01 - 08:00).* |  |
| Zafar, 2015.^144^ | Time of day of operation | *Daytime (07:00 – 17:00) or nighttime (00:00 – 05:59).* |  |

1. None of the real-life studies included any validates measures of fatigue.

**Supplementary Table 2: Measures used to assess fatigue in simulator studies.**

| **Source** | **Operationalization of fatigue** | | **Other measures of fatigue** | |
| --- | --- | --- | --- | --- |
|  |  |  | **Validated** | **Non-validated** |
| Amirian, 2014.^145^ | Pre-call vs. on-call | *Pre-call (08:00) vs. on-call (04:00 on the subsequent night on call).* | Karolinska Sleepiness Scale; Wrist actigraphy. | Sleep diary (time in and out of bed, time of sleep, and number and duration wake episodes). |
| Brandenberger, 2010.^16^ | Pre-call vs. post-call | *Before or after a 12-hour duty period.* | Behrenz and Monga fatigue questionnaire. | Hours slept during call; Caffeine intake during call. |
| Bykanov, 2021.^146^ | Pre-call vs. post-call; Sleep deprived (SD) vs. non-sleep deprived (NSD) | *Baseline vs. morning after end of 24-hour shift with no more than 4 hours sleep per shift.* |  |  |
| Cumpanas, 2020.^147^ | Work hours | *Before or after a 4-hour simulator training session.* |  |  |
| Cumpanas, 2020.^148^ | Pre-call vs. post-call | *Before or after an 18-hour overnight shift.* |  |  |
| Eastridge, 2003.^149^ | Pre-call vs. on-call vs. post-call | *Pre-call (morning before 24-hour in-house call), on-call (morning of call) and post-call (morning after call) – first two time points represent rested state.* |  | Hours slept preceding 24 hours; Quality of sleep; Subjective fatigue levels; Number of hours worked previous 7 days. |
| Ganju, 2012.^150^ | Pre-call vs. post-call | *Before or after 24-hour period of in-house call responsibilities.* | Behrenz and Monga fatigue questionnaire. | Hours slept during call; Caffeine intake during call. |
| Gerdes, 2008.^17^ | Pre-call vs. post-call | *Pre-call (05:30 – 06:30) or post-call (05:30 – 06:30 subsequent night).* | Behrenz and Monga fatigue questionnaire. | Hours slept during call; Caffeine intake during call. |
| Grantcharov, 2001.^151^ | Pre-call vs. post-call; Sleep deprived (SD) vs. non-sleep deprived (NSD) | *Normal working hours or after a night on call with impaired sleep.* |  |  |
| Hegar, 2011.^152^ | Pre-call vs. on-call vs. post-call | *Pre-call, 12 hours, 18 hours, or 24 hours into the call day.* |  | Subjective fatigue levels; Work hours; Sleep patterns. |
| Jakubowicz, 2005.^153^ | Pre-call vs. post-call | *Before or after a 24-hour on-call period.* |  | Sleep interruptions: Hours slept preceding 24 hours; Time from awakening to testing; Quality of sleep and fatigue at time testing; Caffeine, alcohol, and cigarette intake during preceding 24 hours. |
| Kahol, 2008.^11^ | Pre-call vs. post-call | *Before or after a period of night call duties.* | Behrenz and Monga fatigue questionnaire. |  |
| Kahol, 2011.^18^ | Pre-call vs. post-call | *Before or after overnight call.* | Behrenz and Monga fatigue questionnaire. |  |
| Kocher, 2006.^154^ | Sleep deprived (SD) vs. non-sleep deprived (NSD) | *SD = subject out for meal and some sleep deprivation before returning to work in the morning. NSD = subject rested after work and full night’s undisturbed sleep.* |  | Hours slept preceding 24 hours. |
| Leff, 2008.^155^ | Post-call vs follow-up. | *Post-night shift from 20:00 – 08:00 vs. well-rested follow-up week later.* | Epworth Sleepiness Scale. | Questionnaire on estimated total sleep preceding week, hours daytime sleep prior to night shift, length of naps during night shift, quality of sleep, number of interruptions, quantity caffeinated drinks consumed, and cigarettes smoked. |
| Leff, 2010.^156^ | Sleep deprived (SD) vs. non-sleep deprived (NSD) | *At 22:00 (NSD) and every 2 hours until 08:00 the following day (SD).* | Epworth Sleepiness Scale. | Hours slept preceding 24 hours. |
| Schlosser, 2012.^14^ | Pre- call vs. post-call | *Morning ahead of 24-hour call (pre-call), the morning after 24-hour call (post-call) or morning after 24-hour rest (control).* | Stanford Sleepiness Scale; Saliva Cortisol-ELISA; Pupillography. | Hours slept preceding 24 hours. |
| Taffinder, 1998.^157^ | Sleep deprived (SD) vs. non-sleep deprived (NSD); On-call vs control | *Baseline (17:00 – 18:00) or following morning after (1) undisturbed night (NSD, control), (2) sham night on call, disturbed at 00:00, 03:00 and 06:00 (on-call), or (3) a night with no sleep (latter two SD).* |  |  |
| Tsafrir, 2015.^158^ | Pre-call vs. post-call | *Before or after a 24-hour on-call period.* |  | Hours slept preceding 24 hours; Caffeine intake preceding 24 hours. |
| Uchal, 2005.^13^ | Post-call vs. post-work | *Post-call = morning after 24h on-call shift in hospital.*  *Post-work = after work-day from 08:00 – 16:00 with previous undisturbed night spent at home.* | Epworth Sleepiness Scale. | Hours slept preceding 24 hours. |
| Veddeng, 2014.^159^ | Pre-call vs. post-call | *Pre-call = morning (07:30 – 10:30) after a normal period of sleep at home. Post-call =*  *end of overnight shift.* | Karolinska Sleepiness Scale. | Hours slept preceding 24 hours; Caffeine intake preceding 12 hours. |
| Waqar, 2011.^160^ | Work hours | *Before or after live theatre session.* |  |  |
| Whelehan, 2021.^161^ | Pre-call vs. post-call | *Morning of their on-call shift (pre-call) vs. morning after on-call or after first night of shift-work (post-call).* | EEG for sleep latency pre-call and post-call; “Pillow” sleep quality tracker (based on heart rate, body movement, and audible sounds); Pittsburgh Sleep Quality Index; Chalder-Fatigue Scale; Epworth Sleepiness Scale. | Sleep-log journals. |
| Yi, 2013.^162^ | Pre-call vs. post-call | *Before and after 24-hour on-call shift vs. before and after night-float shift.* | Epworth Sleepiness Scale. | Survey reporting fatigue modifiers, such as hours slept, caffeine intake, numbers of steps walked. |

**Supplementary Table 3: Outcome simulator studies between fatigued and non-fatigued surgeons.**

| **Study** | **No. of surgeons** | **Surgical skill (and equipment)** | **Surgical outcome measures (CF: cognitive function, PF: psychomotor function)** | **Results^(A)^** | **Effect fatigue on surgical performance** |
| --- | --- | --- | --- | --- | --- |
| Amirian, 2014.^145^ | 29 surgeons: 9 interns, 11 residents and 9 attendings | Laparoscopic simulation of ectopic pregnancy (*LapSimGyn virtual reality simulator)* | PF*:* blood loss, instrument path length, instrument angular length. | No difference in outcome measures when fatigued. | NA |
| Brandenberger, 2010.^16^ | 14 surgical residents | Laparoscopic proficiency (*ProMIS and FLS simulator)* | CF: cognitive errors.  PF: gesture-level proficiency, hand movement smoothness, instrument movement smoothness. | More cognitive errors, and decrease in gesture-level proficiency, hand movement smoothness and instrument movement smoothness when fatigued. CF was significantly worse at end shift in night-float group compared to day-shift group. | 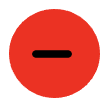 |
| Bykanov, 2021.^146^ | 11 neurosurgery residents | Simulated neurosurgical manipulation (*Patent for invention No. RU2679297C1)* | PF: spatial accuracy (no. registered errors (touching limited frame)). | No difference in outcome measures when fatigued. | NA |
| Cumpanas, 2020.^147^ | 15 surgeons | Virtual reality robotic surgery (*da Vinci surgical simulator)* | PF: instrument collision, excessive force applied to instruments, instruments out of view, economy of motion, drops, master workspace, misapplied energy time. | Decrease PF when fatigued: more instrument collision in 1/3 tasks, more excessive force in 3/3 tasks, more instruments out of view in 3/3 tasks, worse economy of motion in 3/3 tasks, more drops in 2/2 tasks, worse master workspace in 3/3 tasks before vs. after 4h training session. Misapplied energy time was not worse when fatigued. | 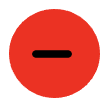 |
| Cumpanas, 2020.^148^ | 20 surgical residents | Virtual reality robotic surgery (*da Vinci Xi platform*) | PF: Instrument collision, excessive force applied to instruments, instruments out of view, economy of motion, drops, master workspace, misapplied energy time. | Decrease in PF when fatigued: decrement in all outcome measures in all 3 task post-shift compared to pre-shift. | 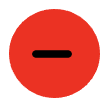 |
| Eastridge, 2003.^149^ | 35 surgical residents | Laparoscopic proficiency (*MIST VR simulations)* | PF: Number of errors, economy of motion. | Increase in number of errors when fatigued. No difference in economy of motion. | 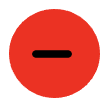 |
| Ganju, 2012.^150^ | 7 neurosurgery residents | Laparoscopic proficiency (*ProMIS and FLS simulator)* | CF: cognitive error (error of judgement).  PF: movement smoothness. | No difference in outcome measures when fatigued. | NA |
| Gerdes, 2008.^17^ | 14 trauma surgical staff: 5 trauma residents and 9 trauma surgeons | Laparoscopic proficiency (*ProMIS and FLS simulator*) | CF: cognitive error (error of judgement).  PF: gesture level proficiency, hand movement smoothness, tool movement smoothness. | Decrement in all CF and PF measures when fatigued. Attending surgeons made 25% fewer cognitive errors than residents when fatigued. PF equally affected in both groups. | 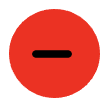 |
| Grantcharov, 2001.^151^ | 14 surgeons | Laparoscopic proficiency *(MIST-VR)* | PF: errors, no. unnecessary movements. | More errors in 2/6 tasks, more unnecessary movements in 2/6 tasks. | 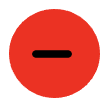 |
| Hegar, 2011.^152^ | 14 surgical participants: 7 interns, 7 residents | Laparoscopic proficiency *(Symbionix Laparoscopic GI Mentor computer system)* | PF: Efficiency (economy of motion, efficiency of cautery), accuracy. | No difference in outcome measures when fatigued.^(B)^ | NA |
| Jakubowicz, 2005.^153^ | 8 surgical residents | Simulated endoscopic sinus surgery (*ES3 software by Lockheed Martin)* | PF: Hazard score (percentage area of each obstacle dissected). | No difference in outcome measures when fatigued. | NA |
| Kahol, 2008.^11^ | 37 trauma surgery and OB/GYN residents | Laparoscopic proficiency *(ProMIS and FLS simulator)* | CF: cognitive errors.  PF: gesture proficiency, hand- and tool-movement smoothness. | Increase in cognitive errors, decrease in gesture proficiency and hand- and tool movement smoothness in cognitive skill dominated exercises when fatigued, but not affected in purely psychomotor tasks. | 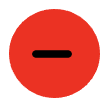 |
| Kahol, 2011.^18^ | 7 surgical residents | Laparoscopic proficiency (*ProMIS and FLS simulator)* | CF: cognitive errors.  PF: hand- and instrument-movement smoothness, gesture proficiency. | Increase in cognitive errors, decrease in gesture proficiency, hand- and instrument-movement smoothness in tasks in which cognitive dimensions were embedded when fatigued, but not in purely psychomotor tasks. | 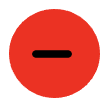 |
| Kocher, 2006.^154^ | 5 surgeons | Laparoscopic proficiency *(MIST-VR)* | PF: No. of errors. | No difference in outcome measures when fatigued. | NA |
| Leff, 2008.^155^ | 13 surgical residents: 7 general surgery, 4 OB/GYN, 2 orthopaedic. | Laparoscopic proficiency *(MIST-VR)* | PF: No. of errors, economy of motion. | No difference in outcome measures when fatigued. | NA |
| Leff, 2010.^156^ | 7 surgical residents | Surgical knot tying *(Bench-top trainer)* | PF: Total movements, total path length. | No difference in outcome measures when fatigued.^(C)^ | NA |
| Schlosser, 2012.^14^ | 38 surgical participants: 19 residents and 19 interns | Laparoscopic proficiency *(LapSim)* | CF: performance score, left – and right instrument path length and angular path in high-fidelity tasks, error score (reversed performance score).  PF: Performance score, cutter- and grasper path length and angular path in low-fidelity tasks, error score (reversed performance score). | Improved performance score, and left- and right instrument path length and angular path in high fidelity task, representing improved cognitive performance.^(D)^ | 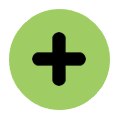 |
| Taffinder, 1998.^157^ | 6 surgical residents | Laparoscopic proficiency *(ICSAD and MIST VR)* | PF: Errors. | Increase in no. of errors when fatigued.^(E)^ | 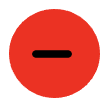 |
| Tsafrir, 2015.^158^ | 26 OB/GYN residents | Laparoscopic proficiency *(Simbionix Lap Mentor simulator)* | PF: Correct hits; Maintaining horizontal view and time horizontal view maintained with 0-degree camera; camera path length, speed, and shots; economy of movement specific instruments; accuracy rates tasks; instrument movement, economy of movement, speed; instrument total, ideal and relevant path length; clipped ducts; lost clips; total clipping attempts; safe retraction; cutting maneuvers general, and without causing injury; total retraction operations, and without overstretch injuries; efficiency of cautery; no. highlighted and nonhighlighted bands cut; time cautery applied without contact, or on nonhighlighted bands; peg transfer (not, forward, reversed); pegs dropped; task completion (complete/incomplete/timed out); recommended performance level (achieved/not achieved); area of accuracy error. | Worse safety (more errors), and lower accuracy rates when fatigued. | 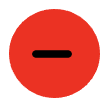 |
| Uchal, 2005.^13^ | 64 surgeons | Laparoscopic proficiency *(MIST-VR)* | PF: Tissue damage, accuracy error, leak rate, goal-directed actions, non-goal directed actions. | No difference in outcome measures when fatigued. | NA |
| Veddeng, 2014.^159^ | 28 gynaecologists | Simulated salpingectomy surgery *(VR SimSurgery)* | PF: Instrument movement length (tip trajectory), blood loss. | No differences in instrument movement length or blood loss when fatigued. | NA |
| Waqar, 2011.^160^ | 7 ophthalmic surgeons | Simulated cataract surgery *(Eyesi simulator)* | PF: lens injury score, odometer score, operating without red reflex. | No difference in outcome measures when fatigued. | NA |
| Whelehan, 2021.^161^ | 20 surgical trainees and consultants | Laparoscopic proficiency *(SIMENDO simulator)* | PF: Errors, economy of motion (path length). | Increased right path length in task in which both technical and cognitive dimensions were imbedded when fatigued. | 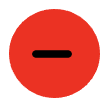 |
| Yi, 2013.^162^ | 9 general surgery residents | Laparoscopic proficiency *(Simbionix Lap Mentor simulator)* | PF: Moves, accuracy, economy of movement, speed. | No difference in outcome measures when fatigued. | NA |

1. Solely uni- or bivariate analyses were performed to analyse the difference in performance between fatigued and non-fatigued surgeons.
2. No difference between 0h and 24h mark in accuracy of efficiency. Only significant difference is lower accuracy in interns at 18-hour mark.
3. No difference in total movements or total path length between first- and final-time sessions. Improvement was shown in total movements and path length between the first and second session but stabilized thereafter.
4. In one low-fidelity task, an improvement was shown in performance score, cutter path-length and angular path, and grasper angular path post-call. According to the authors however, these results were inconsistent in the analysis, and not reproducible.
5. Significant linear trend across sleep conditions (control/non-sleep deprived – on-call – sleep deprived) for error score.

**Supplementary Table 4: Outcome real-life studies between fatigued and non-fatigued surgeons.**

| **Study** | **No. of patients** | **Surgical skill** | **Surgical outcome measures** | **Results (based on solely univariate (UV), or multivariate (MV) analysis)** | **Effect fatigue on surgical performance** |
| --- | --- | --- | --- | --- | --- |
| A-lai, 2022.^36^ | 398 patients | Elective lung surgery | Operative mortality (in-hospital or within 30-days), occurrence complications (further specified), lung infection, prolonged air leak, chylothorax, intraoperative blood loss, transfusion, postoperative and ICU LOS, and intraoperative conversion. | No significant difference in surgical outcome. | NA |
| Ahlsson, 2019.^37^ | 1159 patients | Type A acute aortic dissection (TAAAD) surgery | Intraoperative and 30-day mortality, occurrence any complications, perioperative myocardial injury, postoperative stroke, TIA, arrest, cardiac tamponade or atrial fibrillation, deep sternal wound infection, sepsis, renal replacement therapy, ventilation >48 hours, acute limb ischemia, heart block requiring pacemaker, multisystem organ failure, and reoperation for bleeding. | No significant difference in surgical outcome. | NA |
| Alnajashi, 2020.^38^ | 330 patients | Trauma surgery | Occurrence complications (not further specified). | No significant difference in surgical outcome. | NA |
| Araujo, 2014.^39^ | 819 patients | Elective pancreatico-duodenectomy | 90-day mortality, occurrence overall and high-grade complications (not further specified), rate of anastomotic leak or fistula, positive surgical margins, estimated blood loss, and LOS. | Longer LOS for operations started in the afternoon. (UV) | 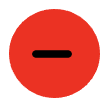 |
| Arnaoutakis, 2020.^40^ | 4197 patients | Type A acute aortic dissection (TAAAD) surgery | 30-day and 5-year post discharge mortality, postoperative CVA, coma, spinal cord ischemia, myocardial ischemia, mesenteric ischemia, acute kidney injury, extension of dissection, hypotension, limb ischemia, respiratory insufficiency, bleeding requiring reoperation, and postoperative LOS. | No significant difference in surgical outcome. | NA |
| Assali, 2006.^41^ | 273 patients | Primary coronary angioplasty | In-hospital and 30-day mortality, in-hospital and 30-day recurrent MI, in-hospital and 30-day stent thrombosis, 30-day CABG, 30-day composite adverse cardiac event (death, MI, target vessel revascularization), and procedural success. | Higher in-hospital mortality and unadjusted composite adverse cardiac event in nighttime group. (UV) Time of treatment was not an independent predictor for 30-day mortality in multiple logistic regression. | 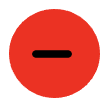 |
| Axtell, 2020.^42^ | 2460 patients | Cardiac surgery | Operative mortality (in-hospital or within 30 days), postoperative transfusion, prolonged ventilation >24 hours, acute renal failure, MI, stroke, deep sternal wound infection, sepsis, atrial fibrillation, reoperation for bleeding, ICU LOS, hospital LOS, and postoperative LOS. | No significant difference in surgical outcome. | NA |
| Aydoğmuş, 2017.^43^ | 91 patients | Pediatric supracondylar humeral fracture surgery | Occurrence postoperative neurovascular complications (not further specified), rate of unsuccessful reduction, poor fixation, induced deformity, and functional loss. | Poor fixation rate was higher in after-hours compared to daytime. (UV) | 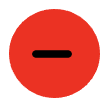 |
| I - Bagrodia, 2012.^44^ | 183 patients | Percutaneous nephrostolithotomy | Occurrence complications (not further specified), LOS, blood transfusion, no. residual fragments, no. 2nd looks, stone free, and multiple access. | No significant difference in surgical outcome. | NA |
| II - Bagrodia, 2012.^44^ | 746 patients | (Robot-Assisted) laparoscopic prostatectomy | Cancer specific mortality, occurrence intraoperative and hospital complications (not further specified), hospital LOS, blood transfusion, estimated blood loss, no. nerve sparing, positive margins, postoperative continence, potency, PSA recurrence, no. drain placement, and no. lymphadenectomy. | No significant difference in surgical outcome. ^(A)^ | NA |
| Bailit, 2006.^45^ | 18939 patients | Unscheduled cesarean delivery | Maternal complications (namely maternal blood transfusion, cystotomy, bowel and urethral injury, postpartum endometritis, wound infection, wound hematoma, ileus, cesarean hysterectomy, postoperative ventilation, readmission, maternal ICU admission, total maternal complications, and maternal death), neonatal complications (namely neonatal ICU admissions, cord pH <7.00, base excess <-12, brachial plexus injury, skull fracture, facial nerve palsy, clavicular fracture, other fractures, fetal lacerations, neonatal seizures and infant death). | No significant difference in surgical outcome. ^(B)^ | NA |
| Barinaga, 2017.^46^ | 441 patients | Hip fracture surgery | In-hospital mortality, LOS, estimated blood loss, 30-day readmission rates, and postoperative hematocrit levels. | No significant difference in surgical outcome. | NA |
| Becker, 2019.^47^ | 350 patients | Orthotopic liver transplantation | Death within initial stay, 30-day, 90-day, and one-year patient and graft survival, death-censored graft survival, overall graft survival, primary non-function, no. biopsy proven acute rejections, occurrence total, hemorrhage, vascular, biliary, wound-healing, and GI complications requiring reoperation (further specified), occurrence other complications (not further specified); ICU LOS, hospital LOS, no. re-transplantations, frequencies of endoscopic retrograde cholangiopancreatography, no. and length of re-admissions after initial hospital discharge, and peak serum values of alanine transaminase/aspartate transaminase. | No significant difference in surgical outcome. | NA |
| Bekelis, 2018.^48^ | 4700 patients | Unruptured cerebral aneurysm surgery | In-hospital mortality, hospital LOS, and rate of discharge to a facility (any other than patient’s home). | No significant difference in surgical outcome. | NA |
| Bianco, 2021.^49^ | 3429 patients | Cardiac surgery | Operative mortality, 30-day, one-year, and five-year postoperative mortality, survival and readmission, overall postoperative mortality and readmission, blood transfusion, ventilation >24 hours, deep sternal wound infection, acute renal failure, sepsis, pneumonia, permanent stroke, new-onset atrial fibrillation, ICU LOS, hospital LOS, and reoperation. | Lower ICU LOS in nighttime group. (UV) | 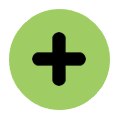 |
| de Boer, 2018.^50^ | 591 patients | Transplantation surgery | Procurement-related vessel and organ surgical injury. | More procurement-related surgical injuries in nighttime group compared to daytime group. (MV) | 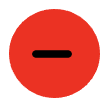 |
| Boscà, 2019.^51^ | 439 patients | Liver transplantation surgery | Overall survival, occurrence vascular and biliary complications (not further specified), and no. early reoperation for postoperative bleeding. | No significant difference in surgical outcome. | NA |
| Canal, 2020.^52^ | 9224 patients | Appendectomy | In-hospital mortality, occurrence of complications (not further specified), total and postoperative LOS, ICU LOS, need for reintubation, and discharge disposition. | Higher postoperative LOS in the evening and higher need for reintubation in the morning. (UV) | 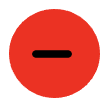/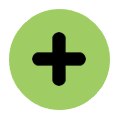 |
| Capello, 2008.^53^ | 381 patients | Robot-Assisted radical prostatectomy | Occurrence total, surgical and medical complication (further specified), estimated blood loss, positive margins, and PSA recurrence. | No significant difference in surgical outcome. | NA |
| Chacko, 2011.^54^ | 767 patients | Hip fracture surgery, including Dynamic Hip System (DHS), Hemiarthroplasty, or an intramedullary nail | One-month, one-year and two-year mortality, in-house pneumonia, PE, cardiac event, DVT, GI bleed and infection, post-discharge PE, DVT and infection, nonunion, implant failure, refracture, hospital LOS, ICU LOS, estimated blood loss, no. transfusions, and reoperation. | For DHS, higher blood loss and higher no. transfused units during after-hours. (UV) | 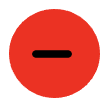 |
| Chan, 2018.^55^ | 367 patients | Hip fracture surgery | 30-day mortality, and occurrence complications related to surgical procedure (not further specified). | No significant difference in surgical outcome. | NA |
| Chang, 2008.^56^ | 159 patients | Laparoscopic assisted vaginal hysterectomy | Occurrence complications (not further specified), hospital LOS, estimated blood loss, blood transfusion, flatulence relief time, IV-fluid injections >2 days, and shift serum haemoglobin and serum haematocrit. | No significant difference in surgical outcome. | NA |
| Chen, 2020.^57^ | 15767 patients | Video-assisted thoracoscopic pulmonary resection | Occurrence intraoperative complications (further specified), and postoperative LOS (in case of complication). | Higher occurrence intraoperative complications during nighttime for lobectomy and segmentectomy. (MV) | 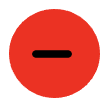 |
| Chiu, 2019.^58^ | 108 patients | Posterior Spinal Fusion surgeries | Occurrence complications (not further specified), hospital LOS, estimated blood loss, blood transfusion, intraoperative pH, HCO3- and lactate, postoperative haemoglobin level, haemoglobin drift, PCA morphine usage, postoperative Cobb angle, correction rate, side bending flexibility, and side bending correction index. | No significant difference in surgical outcome. ^(C)^ | NA |
| Chu, 2011.^59^ | 4047 patients | Cardiac surgery | Hospital mortality, occurrence any complication (further specified), postoperative MI, respiratory failure, sternal dehiscence, mediastinitis, septicaemia, new renal failure, arrest/permanent pacemaker, reoperation for bleeding, stroke/delirium, postoperative intra-aortic balloon pump, ICU LOS, and hospital LOS. | Higher incidence septicemia in the 0-3 hours’ sleep group. Shorter LOS in the 0-6 hours’ vs >6 hours’ sleep group. (UV) | 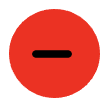/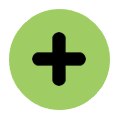 |
| Cook, 1997.^60^ | 102 patients | Urgent and emergency surgery | In-hospital mortality | No significant difference in surgical outcome. | NA |
| Coumbe, 2011.^61^ | 4714 patients | CABG Surgery | Operative mortality (death <30 days of operation due to any cause or death >30 days directly related to complication of the cardiac surgery), occurrence any complication (not further specified), stroke, ventilation >48 hours, and reoperation for bleeding. | Higher incidence reoperation for bleeding, postoperative stroke, prolonged ventilation, and any complication in patients operated after regular work hours. (UV) | 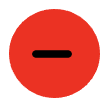 |
| Dalton, 2016.^62^ | 230 patients | Urgent and emergency surgery | Mortality, failure to rescue (death after major complication), occurrence any or total complications (further specified), no. ventilator days, organ space infection, SSI, wound dehiscence, postoperative bleeding, acute kidney injury, postoperative MI, dysrhythmia, ICU LOS, postoperative LOS, hospital LOS, estimated blood loss, intraoperative intravenous fluids, creation of enterostomy, abdomen left open, not extubated in OR, unplanned return to OR, and readmission. | After controlling for confounders, surgical care at night was a potent predictor of mortality but had little impact on morbidity. (MV) Higher dysrhythmia rates at night. (UV) | 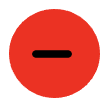 |
| Ellman, 2004.^63^ | 6751 patients | Cardiac surgery | In-hospital mortality, occurrence operative, neurological, renal, infectious, or pulmonary complications (further specified), hospital LOS, and no. blood products. | No significant difference in surgical outcome. | NA |
| Ellman, 2005.^8^ | 7323 patients | Cardiac surgery | In-hospital mortality, occurrence operative, neurological, renal, infectious, or pulmonary complications (further specified), hospital LOS, and no. blood products. | No significant difference in surgical outcome. | NA |
| Eskesen, 2018.^64^ | 9136 patients | Abdominal surgery | 30-day mortality, occurrence any postoperative complication (further specified), transfusion, SSI, superficial SSI, deep SSI, organ-space SSI, wound dehiscence, systemic sepsis, CVA, DVT, PE, cardiac arrest, MI, pneumonia, unplanned intubation, failure to wean of ventilation >48 hours, progressive renal insufficiency, acute renal failure, UTI, postoperative LOS, hospital LOS, and no. and severity of intraoperative adverse events (iAEs), defined as an injury caused by medical management. | Shorter postoperative and hospital LOS in nighttime group. Higher 30-day mortality in nighttime group. Higher occurrence any complication in evening group, least in daytime group. Higher transfusion rate and more DVTs in evening group. More wound dehiscence, unplanned intubation, failure to wean off ventilator for >48 hours and acute renal failure in nighttime group. Less UTIs during the night. (UV) | 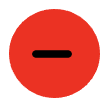/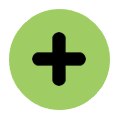 |
| Fechner, 2008.^65^ | 260 patients | Kidney transplantation surgery | Occurrence total complications requiring reoperation (further specified), urethral and vascular stenosis/leakage, graft vein thrombosis, hematoma, nephrectomy for ischemia, graft failure, and graft function. | Nighttime operation was associated with a higher risk of long-term-graft-failure. Higher total complication and vascular stenosis/leakage during nighttime. (UV) | 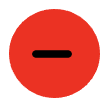 |
| Fernandes, 2016.^66^ | 330 patients | Urgent colorectal surgery | 30-day mortality, occurrence medical or surgical postoperative complication (not further specified), wound infection, anastomotic dehiscence, abscess, hospital LOS, ICU hospitalization, and no. relaparotomy. | Shorter hospital LOS in nighttime group. (UV) | 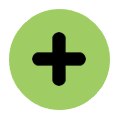 |
| Gabriel, 2018.^67^ | 46196 patients | Emergency non-cardiac, non-obstetric surgeries undergoing general anesthesia | Perioperative mortality. | No significant difference in surgical outcome. | NA |
| Gasser, 2020.^68^ | 319 patients | Type A Aortic Dissection (TAAD) surgery | Death before cardiopulmonary bypass, death within 24 hours, 30-day mortality, postoperative neurological injury (further specified), postoperative malperfusion syndrome, extracorporeal circulatory membrane oxygenation, tracheotomy, ICU LOS, in-hospital LOS, postoperative haemofiltration, revision due to bleeding, and reoperation during follow-up. | No significant difference in surgical outcome. | NA |
| George, 2011.^69^ | 27118 patients | Heart and lung transplantation surgery | 30-day, 90-day and one-year mortality, postoperative CVA, airway dehiscence, new onset dialysis, drug-treated infection, need for cardiac reoperation, pacemaker placement, need for other (non-cardiac) surgery, and total LOS. | Nighttime lung transplantation surgery had a higher rate of airway dehiscence. (UV) ^(D)^ | 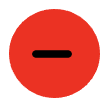 |
| Goertz, 2021.^70^ | 157 patients | Microsurgical clipping for aneurysmal subarachnoid hemorrhage | In-hospital mortality, intraoperative rupture, treatment-related cerebral infarction, cerebral vasospasm, overall cerebral infarction, unfavorable outcome (based on modified Rankin Scale score 3-6) at discharge and at 6-month follow-up, and aneurysm occlusion. | No significant difference in surgical outcome. | NA |
| Govindarajan, 2015.^71^ | 38978 patients | Elective surgery: cholecystectomy, gastric bypass, colon resection, CABG, coronary angioplasty, knee / hip replacement, hip fracture repair, hysterectomy, spinal surgery, craniotomy, and lung resection | Death within 30 days, occurrence postoperative complications within 30-days (further specified), hospital LOS, readmission within 30 days, and composite outcome of death, readmission, or complication within 30 days. | No significant difference in surgical outcome. | NA |
| Guidry, 2016.^72^ | 21985 patients | Emergency, cardiothoracic, vascular or transplant surgery | 30-day mortality, occurrence ACS NSQIP-defined serious complications (not further specified), and no. postoperative infections. | No significant difference in surgical outcome. | NA |
| Guo, 2019.^73^ | 443 patients | Kidney transplantation surgery | Recipient and graft survival up to 48 months, occurrence total surgical, vascular, and urologic complications (further specified), occurrence wound complications (not further specified), hematoma around graft, nosocomial infections, hospital LOS, ICU LOS, reoperation, graft function, delayed graft function, and acute total, biopsy proven, and clinically inferred rejection. | Longer ICU LOS in the nighttime group. (UV) | 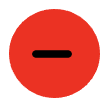 |
| Halldorson, 2009.^74^ | 390 patients | Liver transplantation surgery | One-year and three-year patient and graft survival, hepatic artery thrombosis, bile leakage/stenosis, one-unit packed RBCs or fresh frozen plasma in 48 hours, transplant-related reoperation, reoperation for bleeding, primary non-function, and initial LOS. | One-year patient and graft survival and three-year patient survival were higher with >2 days between transplants. (UV) | 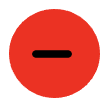 |
| Halliday, 2019.^75^ | 8816 patients | Liver transplantation surgery | 30-day, one-year and three-year post-transplant graft or transplant failure. | No significant difference in surgical outcome. | NA |
| Halvachizadeh, 2019.^76^ | 31692 patients | Orthopaedic trauma surgery | In-hospital mortality, and occurrence general, intraoperative, or postoperative complications (further specified). | Mortality and general complication rate were higher in surgeries in afternoon or night vs. in the morning. (MV) | 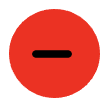 |
| Haynes, 1995.^77^ | 6371 operations | General, vascular, oncologic, pediatric, transplantation surgery | Occurrence complications (not further specified). | No significant difference in surgical outcome. | NA |
| Heller, 2017.^78^ | 3534 patients | Elective cardiac surgery | In-hospital/30-day mortality, total complications (further specified), postoperative atrial fibrillation, adverse EKG changes, SSI, stroke, prolonged mechanical ventilation, renal or multisystem organ failure, reintubation after initial extubation, bleeding requiring reoperation, last epinephrine infusion dose, length of initial intubation, hospital LOS, and ICU LOS. | No significant difference in surgical outcome. | NA |
| Hsu, 2015.^79^ | 148004 patients | First time implantable cardioverter-defibrillator (ICD) procedures (recipients) | In-hospital mortality, occurrence any, major and minor complication (further specified), lead dislodgement, pneumothorax, cardiac arrest, coronary venous dissection, pericardial tamponade, infection requiring antibiotics, cardiac perforation, TIA/stroke, MI, urgent cardiac surgery, hemothorax, peripheral embolus, valve injury, hematoma, drug reaction, conduction block, set screw problem, venous obstruction, peripheral nerve injury, and hospital LOS. | Greater odds hospital stay >1 day and of any complication in afternoon and evening group compared with morning group. (MV) Higher risk any major complication, and more lead dislodgement, cardiac arrest, infections requiring antibiotics, TIA/stroke, peripheral embolus, and hematoma. (UV) | 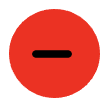 |
| Ishiyama, 2019.^80^ | 1961 patients | Laparoscopic colectomy for colorectal cancer | 30-day mortality, 5-year overall and disease-free survival, total general and intraoperative complications (further specified), vascular injury, adjacent organ injury, wound infection, anastomotic leakage, ileus, intra-peritoneal abscess, pneumonia, enteritis, perforation, anastomotic bleeding or stenosis, bleeding, cardiovascular system complication, estimated blood loss, hospital LOS, median proximal and distal resection margin, and conversion to open surgery. | Higher total intraoperative complications and higher intraoperative organ injury, lower no. intra-peritoneal abscess in and smaller median proximal resection margin in afternoon group compared with morning group. (UV) | 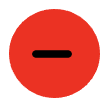/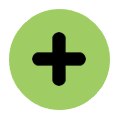 |
| Karagoz, 2022.^81^ | 124 patients | Femoral neck fracture surgery | Avascular necrosis, implant failure, deep infection, estimated blood loss, revision rate, anteroproximal (AP) malreduction, AP + lateral malreduction, lateral malreduction, good reduction, and Harris Hip Score (clinical function). | Higher estimated blood loss in after-hours group. (UV) | 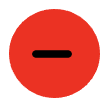 |
| Kelz, 2008.^82^ | 141428 patients | Nonemergent general (musculoskeletal system, cardiothoracic system, digestive system, heme system, endocrine system) and vascular surgery | 30-day mortality, total complications (stated in study as 30-day morbidity, further specified), MI, cardiac arrest, coma, CVA/stroke, DVT/thrombophlebitis, graft/prosthesis failure, systemic sepsis, acute renal failure, renal insufficiency, UTI, failure to wean off ventilation >48 hours, pneumonia, PE, unplanned intubation, wound dehiscence, superficial and deep wound infection, bleeding requiring >4 units RBCs, and peripheral nerve injury. | Elevated risk of morbidity in evening and nighttime group compared to daytime group. (MV) More systemic sepsis, DVT/thrombophlebitis, cardiac arrest, MI, pneumonia, unplanned intubation, PE, failure to wean >48 hours postoperative, UTI, wound dehiscence, and superficial as well as deep wound infection in evening/nighttime group. (UV) | 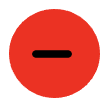 |
| Kelz, 2009.^83^ | 56920 patients | Nonemergent and emergent general and vascular surgical procedures | 30-day mortality, total complications (stated in study as 30-day morbidity, further specified), MI, cardiac arrest, coma, CVA/stroke, DVT/thrombophlebitis, graft/prosthesis failure, systemic sepsis, acute renal failure, renal insufficiency, UTI, failure to wean off ventilation >48 hours, pneumonia, PE, unplanned intubation, wound dehiscence, superficial and deep wound infection, bleeding requiring >4 units RBCs, and peripheral nerve injury. | Increased risk mortality nonemergency cases overnight. Elevated morbidity risk for operations starting between 09:30 – 11:30 and after 17:30, with strongest risk for operations starting between 21:30 – 07:30, mostly as result of elevated risk of morbidity in emergency cases. (MV) More pneumonia, unplanned intubation, failure to wean ventilation, acute renal failure, UTIs, DVT/thrombophlebitis, graft prosthetic failure, >4 units packed RBCs, and systemic sepsis in nonemergency cases during the night. For emergency cases, lower risk PE in the night. (UV) | 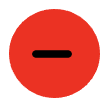/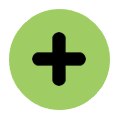 |
| Kienzl-Wagner, 2013.^84^ | 873 patients | Kidney transplantation surgery | Overall (one year and five-year) patient and graft survival, occurrence surgical complications (further specified), acute rejection (clinical need for pulsed steroid treatment or biopsy-proven rejection), and delayed graft function. | No significant difference in surgical outcome. | NA |
| Kim, 2022.^85^ | 1458 patients | Tissue-expander-insertion operation for breast reconstruction. | Occurrence total postoperative complications (further specified), composite major complications (defined as reoperation and/or readmission and premature removal of tissue expander), infection, seroma, hematoma, wound problem, unplanned reoperation, unplanned readmission due to complications, premature removal tissue expander, hospital LOS, and amount consumption additional analgesics (Ketorolac and Demerol). | Higher overall complication, infections, wound problems, reoperations, major complications, and premature removal of tissue expanders. (MV) Higher additional analgesics use, and longer LOS in nighttime group. (UV) | 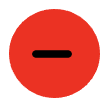 |
| Koltka, 2018.^86^ | 522 patients | Emergency laparoscopic cholecystectomy and appendectomy | Mortality, occurrence intraoperative and postoperative complications (not further specified), and readmission to hospital for late-term complications. | Higher occurrence any intraoperative complications in nighttime group. (MV) | 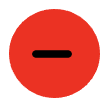 |
| Kork, 2018.^87^ | 247475 patients | In-patient surgery | Postoperative mortality. | Mortality odds ratio lowest in the morning (08:00 – 11:00) and highest in afternoon (13:00 – 17:00). (MV) | 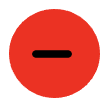 |
| Larsen, 2017.^88^ | 294 patients | Intramedullary nailing (reamed and locked) after tibial shaft fracture | Occurrence complications (further specified), orthopaedic damage (defined as femur fracture, collum femoris fracture, ankle fracture, and/or knee ligament injury), Knee injury and Osteoarthritis Outcome Score (KOOS), and hospital LOS. | Longer hospital LOS when surgery is performed at night by trainees (but not when performed by trauma surgeons). (MV) | 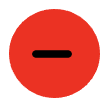 |
| Lee, 2013.^89^ | 454 patients | Elective microsurgical reconstruction surgery using free-tissue transfer | Total minor complications (further specified), flap total, partial and threatened loss, re-exploration due to pedicle thrombosis, wound infection, wound dehiscence, hematoma, seroma formation, hospital LOS, and success rate. | Higher rates of total flap loss and re-exploration, and lower success rate (flap salvage rate) in after-hours group. (MV)  Longer LOS in after-hours group. (UV) | 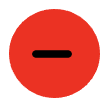 |
| Li, 2018.^90^ | 6548 cases (5183 patients) | Total joint arthroplasty surgery (total hip arthroplasty (THA) and total knee arthroplasty (TKA)) | Occurrence systemic and arthroplastic complications (further specified), acute infection, hospital LOS, and estimated blood loss. | Higher incidence of arthroplastic complications in fourth-round cases. (MV) Longer LOS in fourth-round cases. (UV) | 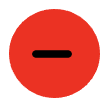 |
| Lim, 2015.^91^ | 2334 patients | Neck of femur fracture surgery | Composite adverse event (defined as mortality, readmission within 30 days or ICU admission), and hospital LOS. | No significant difference in surgical outcome. | NA |
| Lonze, 2010.^92^ | 578 patients | Liver transplantation surgery | 7-day mortality, occurrence any, vascular, wound, biliary and other complications (further specified), postoperative sepsis, hospital LOS, ICU LOS, intraoperative blood product usage, packed RBCs, fresh frozen plasma, and primary graft nonfunction. | Higher risk 7-day mortality nighttime group compared to daytime group. (MV) | 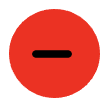 |
| Lu, 2017.^93^ | 383 patients | Partial liver resection surgery | Occurrence other and classification of complication (not further specified), wound infection, postoperative pneumonia, hydrothorax, peritoneal effusion, intra-abdominal infection, intraperitoneal bleeding after hepatectomy, biliary fistula, liver failure, renal dysfunction, hospital LOS, ICU LOS, and estimated blood loss. | No significant difference in surgical outcome. | NA |
| Lu, 2020.^94^ | 231 patients | Hepatic resection for hepatocellular carcinoma (HCC) | Overall survival, estimated blood loss, blood transfusion, packed RBCs, and surgical margin. | No significant difference in surgical outcome. | NA |
| Mehra, 2020.^95^ | 71 patients | Emergency deceased donor renal transplants surgery | Patient and graft survival, occurrence vascular and urologic complications (not further specified), delayed graft function, slow graft function, and creatinine at 1-year post-transplant and at last follow-up. | Higher creatinine at last follow-up in day-time surgery patients. (UV) | 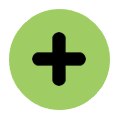 |
| Miyahara, 2021.^96^ | 1189 patients | Elective posterior thoracolumbar spine surgery | Total overall, major, and minor perioperative complications (further specified), SSI, postoperative hematoma, paralysis, and nerve root injury, incidental dural tear, and intraoperative blood loss. | Post-nighttime was risk factor of dural tear in complex cases. (MV) | 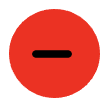 |
| Mönttinen, 2021.^97^ | 1198 patients | Appendectomy for acute appendicitis | 30-day mortality, total complications (further specified), occurrence other complications (not further specified), occurrence Clavien-Dindo I-II and III-IV (not further specified), superficial and organ/space SSI, ileus, bleeding, pneumonia, hospital LOS, intraoperative blood loss, and conversion to open surgery. | Shorter hospital LOS in nighttime group. (UV) | 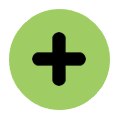 |
| Narayan, 2020.^98^ | 196 patients | Emergency Type A Aortic Dissection (TAAD) repair surgery | 30-day mortality, occurrence postoperative neurological deficit (not further specified), low output syndrome, and re-exploration for bleeding. | No significant difference in surgical outcome. | NA |
| Nasseri, 2022.^99^ | 210 patients | Non-emergent robotic colorectal surgeries | Occurrence intraoperative and postoperative complications (further specified), total GI complications (further specified), abdominal/pelvic abscess, anastomotic leak, ileus/small bowel obstruction, SSI, hospital LOS, estimated blood loss, intraoperative transfusions, conversion to open surgery, and 30-day admission rate. | Longer LOS in afternoon group. (UV) | 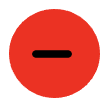 |
| Ndegbu, 2019.^100^ | 267 patients | Emergency abdominal surgery | Mortality, LOS, and re-operation. | No significant difference in surgical outcome. | NA |
| Okkaoglu, 2021.^101^ | 150 patients | Supracondylar humeral fractures Gartland type 3 (completely displaced fracture with an intact periosteal hinge) | Lateral capitellohumeral angle, Baumamn angle and anterior humeral line on postoperative early radiographs (representing reduction quality), and open reduction rate. | No significant difference in surgical outcome. | NA |
| Orman, 2012.^102^ | 94768 patients | Liver transplantation surgery | 30-day, 90-day and 1-year patient and graft survival. | No significant difference in surgical outcome. | NA |
| Özdemir-van Brunschot, 2016.^103^ | 4519 patients | Renal transplantation surgery | Long-term graft survival, total pure technical graft failure (graft loss within 10 days after surgery without signs of (hyper)acute rejection, excluding cases of primary nonfunction (PNF) and non-viable kidney (NVK)), pure technical failure due to vascular, urological or other problems, nonimmunological graft failure (graft loss within 10 days without signs of (hyper)acute rejection, including PNF and NVK), PNF, NVK, and acute rejection with graft loss within 10 days. | Lower incidence of pure technical graft failure in nighttime group compared to daytime group. (MV) Lower incidence technical failure due to vascular problems, and technical failure due to other problems in nighttime group. (UV) | 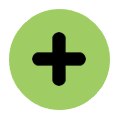 |
| Patel, 2014.^104^ | 340 patients | Intramedullary nailing (IMN) of a diaphyseal femur fracture | Post-operative difference in femoral version (DFV) and femoral length (DFL) between fixed and uninjured sides. | Nighttime surgery (23:00 – 02:59) was predictive of less DFV. (MV) | 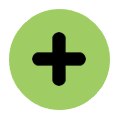 |
| Patel, 2018.^105^ | 25874 patients | Appendectomy | Occurrence complications within 30-days after appendectomy (further specified). | No significant difference in surgical outcome. ^(E)^ | NA |
| Patella, 2017.^106^ | 420 patients | Elective lung resection surgery | In-hospital mortality, occurrence cardiopulmonary and major complications (further specified), and hospital LOS | No significant difference in surgical outcome. | NA |
| Peled, 2011.^107^ | 9944 patients | Non-elective caesarean section | Maternal complications (namely any, postpartum endometritis, wound infection, immediate postpartum haemorrhage, need for haemotransfusion, bladder injury, bowel injury, hospital LOS, prolonged (>7 days) hospitalization), and neonatal complications (namely any, 5-min APGAR <7, umbilical artery pH <7, NICU admission, occurrence neurological morbidity (further specified), clavicular fracture, other fracture). | Higher rate of maternal morbidity (any maternal complication) in night-time group. (MV) More endometritis, postpartum haemorrhage, haemotransfusion, prolonged hospitalization and hospital LOS in night-time group. (UV) No difference in neonatal morbidity. | 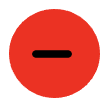 |
| Peskun, 2012.^108^ | 633 patients | Total hip and knee arthroplasty (THA and TKA) | Occurrence medical complication (yes/no, not further specified), intraoperative arterial and nerve injury, periprosthetic fracture, postoperative MI, PE, superficial and deep prosthetic infection, intestinal ileus, UTI, SF-12 physical and mental scores (general health questionnaire scores), WOMAC scores (evaluation condition patients with knee/hip arthritis), component inclination angle >45 degree in THA, femoral component valgus/varus angle >3 degree in THA, and tibial component valgus/varus angle >3 degree in TKA). | No significant difference in surgical outcome. | NA |
| Phatak, 2014.^109^ | 356 patients | Nonelective laparoscopic cholecystectomy | Mortality, overall 30-day complication (further specified), bile leak/biloma, common bile duct injury, retained stone, organ space abscess, superficial SSI, pneumonia, bleeding requiring reoperation, total and postoperative LOS, conversion to open rate, and readmissions related to 30-day surgical complication. | Increased risk of complications in nighttime group. (MV) Shorter total and postoperative LOS in nighttime group. (UV) | 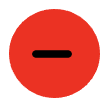/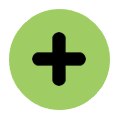 |
| Qiu, 2018.^110^ | 698 patients | Acute Type A Aortic Dissection (TAAD) surgery | In-hospital mortality, occurrence postoperative cardiac complications (further specified), postoperative acute kidney injury, psychonosema, pneumonia, hypothermic cardiac arrest, continuous renal replacement therapy, stroke, paraplegia, hepatic hypofunction, GI bleeding, tracheotomy, ICU LOS, estimated blood loss, repeat thoracotomy, reoperation related aorta, and reintubation. | Higher in-hospital mortality in nighttime group. (MV) Higher rate of reintubation, and higher rate of continuous renal replacement therapy in nighttime group. (UV) | 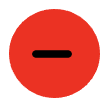 |
| Rashid, 2013.^111^ | 194 patients | Dynamic Hip Screw (DHS) fixation for inter-trochanteric fracture | 30-day mortality, occurrence postoperative complications (not further specified), hospital LOS, estimated blood loss, need for repeat surgery due to technical error, postoperative ambulation weight baring status, and adequacy of fixation (Tip apex index). | No significant difference in surgical outcome. | NA |
| Ricci, 2009.^112^ | 203 patients | Intramedullary nail fixation of femoral and tibial shaft fractures | Healing problems (defined as nonunion, delayed union or malalignment), deep infection, removal of painful hardware, presence of painful hardware, unplanned reoperation, and radiation exposure. | More removal of painful hardware in femoral fracture group during after- hours compared to daytime. (MV) More unplanned reoperations in after-hours group. (UV) | 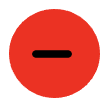 |
| Rogers, 2020.^113^ | 8261 patients | Paediatric liver transplantation surgery | 7-day, 30-day, 90-day and one-year patient and graft survival. | No significant difference in surgical outcome. | NA |
| Rothschild, 2009.^114^ | 4471 surgical patients | Elective surgical procedures (cardiac, general, neurosurgery, orthopaedic, thoracic, vascular, other) | Occurrence total, SSI-, bleeding-, organ/visceral injury- and wound failure-related, and other complications (further specified). | Among post nighttime cases, there was a higher rate of total complications with 6 hours or less of sleep opportunity compared to >6 hours. (MV)  No difference in complications between procedures performed the day after surgeon worked at some point during the night vs. control cases not preceded by nighttime work. | 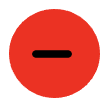 |
| Schieman, 2008.^115^ | 270 patients | Anterior resection for rectal cancer | In-hospital mortality, occurrence intraoperative and long-term complications (further specified), occurrence postoperative and major complications (not further specified), leak/abscess rate, hospital LOS, estimated blood loss, positive margin status, and local and systemic cancer recurrence. | No significant difference in surgical outcome. | NA |
| Schuster, 2018.^116^ | 611 patients | Emergency surgery | In-hospital mortality, wound dehiscence, superficial/deep and organ space SSI, pneumonia, ventilation >48 hours, PE, acute renal failure, postoperative dialysis, UTI, stroke, cardiac arrest, MI, DVT, sepsis, hospital LOS, estimated blood loss, need for postoperative transfusion, unplanned return to OR, fascia closed after laparotomy, ostomy created after colon resection, and discharge disposition. | Shorter hospital LOS in patients of fatigued surgeons. (UV) | 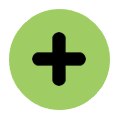 |
| Seklehner, 2016.^117^ | 469 patients | Ureteroscopy | Complete stone removal, partial stone removal, unsuccessful ureteroscopy, ureteral perforations, and ureteral avulsions. | Lower rates complete stone removal and higher rats of unsuccessful ureteroscopy after 12 hours compared to within first 12 hours. (MV) | 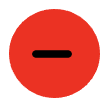 |
| Seow, 2004.^118^ | 322 patients | Kidney transplantation surgery | Occurrence surgical complications (further specified). | No significant difference in surgical outcome. | NA |
| Sessler, 2011.^119^ | 32001 patients | Elective general surgery | 30-day mortality, and occurrence in-hospital complications (not further specified). | No significant difference in surgical outcome. | NA |
| Shah, 2022.^120^ | 1001 patients | Laparoscopic appendectomy | Mortality, bleeding, bowel injury, SSI, SSI location, SSI infection treated by antibiotics or interventional radiological/surgical drainage and antibiotics, hospital LOS, reoperation, conversion to laparotomy, and readmission. | Longer hospital LOS during nighttime group (19:00 - 01:00). (MV) | 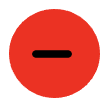 |
| Sharpe, 2013.^121^ | 869 patients | Elective hernia repair surgery (inguinal and ventral), cholecystectomies and intestinal operations. | 30-day mortality, occurrence total, major and minor surgical complications (further specified), and readmission within 30 days. | No significant difference in surgical outcome. | NA |
| Shaw, 2012.^122^ | 633 patients | Kidney transplantation surgery | Patient and death-censored graft survival over course one year, occurrence any, wound, vascular, urologic, GI and bleeding complication (further specified), hospital LOS, ICU LOS, and incidence delayed graft function. | a decrease in  vascular complications in the nighttime cohort, an increase in urologic  complications on subgroup analysis in the 3 AM to 6 AM cohort, and the  12 AM to 3 AM subgroup had the greatest odds of any complication  No significant difference in surgical outcome.  a decrease in  vascular complications in the nighttime cohort, an increase in urologic  complications on subgroup analysis in the 3 AM to 6 AM cohort, and the  12 AM to 3 AM subgroup had the greatest odds of any complication  a decrease in  vascular complications in the nighttime cohort, an increase in urologic  complications on subgroup analysis in the 3 AM to 6 AM cohort, and the  12 AM to 3 AM subgroup had the greatest odds of any complication | NA |
| Siada, 2017.^123^ | 866 patients | Laparoscopic cholecystectomy | Mortality, occurrence complications (further specified), LOS, and conversion to open surgery. | Higher conversion to open surgery rate and longer LOS in daytime group. (UV) | 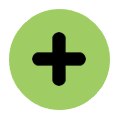 |
| Sugünes, 2019.^124^ | 215 patients | Kidney transplantation surgery | Overall patient and graft survival, occurrence intraoperative, postoperative, and Clavien-Dindo I, II, IIIa, IIIb, IVa, IVb and V complications (yes/no; not further specified), renal artery stenosis or aneurysm, renal vein thrombosis, iliac artery stenosis, renal anastomotic leak, renal pole infarct, coeliac trunk stenosis, haematoma, haemorrhage, urinary leak, urethral necrosis or stricture, urethral stent complication, bladder outflow obstruction/blood clot retention, lymphocele, seroma, wound dehiscence, impaired wound healing, wound infection, acute rejection, delayed graft rejection, and serum creatinine 1, 4, 24 and 60 weeks after transplantation. | Higher overall patient survival in night-time recipients. (UV) | 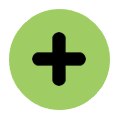 |
| Sun, 2022.^125^ | 498234 patients | Surgical procedures performed by one attending surgeon | Binary composite outcome measure (value of 1 if in-hospital death or occurrence major complication (yes/no; further specified), in-hospital mortality, and occurrence major or minor complication (further specified). | No significant difference in surgical outcome. | NA |
| Switzer, 2013.^126^ | 859 patients | Hip fracture fixation | 30-day mortality, occurrence any complications (further specified), intraoperative fracture, MI, non-MI cardiac event, CNS event, stroke, pneumonia, UTI, wound infection, and bleeding requiring >3 RBC units. | No significant difference in surgical outcome. | NA |
| Tan, 2009.^127^ | 18597 patients | CABG Surgery | In-hospital mortality, and composite morbidity outcome (defined as in-hospital death, acute postoperative MI, neurologic morbidity (focal or global neurologic deficits or death without awakening, serious infectious morbidity (sepsis syndrome or septic shock), new-onset renal failure requiring dialysis, or postoperative ventilatory support >72h). | No significant difference in surgical outcome. | NA |
| Tessler, 2018.^128^ | 7362 patients | Emergency surgery for all specialties except ophthalmology | 30-day mortality. | No significant difference in surgical outcome. | NA |
| Thomas, 2012.^129^ | 481 patients | Pulmonary lobectomy for benign and malignant disease | Occurrence postoperative complications (further specified), and hospital LOS. | Total number of hours a surgeon operated per day is predictor of complication occurrence and increased LOS. (MV) | 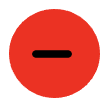 |
| Treacy, 2022.^130^ | 179 patients | Kidney transplantation surgery | Occurrence Clavien-Dindo and major (Clavien-Dindo >2) complications (yes/no; further specified), acute graft rejection, delayed graft function, time before postgraft diuresis, and number of postgraft dialysis. | No significant difference in surgical outcome. | NA |
| Tu, 2021.^131^ | 117 patients | Potentially radical esophagectomy | 90-day mortality, overall survival, disease-free survival, occurrence respiratory and cardiac complications (further specified), anastomotic leak, chylothorax, hoarseness, postoperative LOS, estimated blood loss, intraoperative transfusions, positive resection margins, no. harvested lymph nodes, and no. stations harvested lymph nodes. | Higher occurrence respiratory complications, higher 90-day mortality. (UV) Lower survival, and shorter disease-free survival in after-hours group. (MV) | 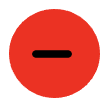 |
| Tu, 2023.^132^ | 321 patients | Elective unilateral total knee arthroplasty | Total postoperative complications (further specified), postoperative delirium and vomiting, anterior knee pain, aseptic loosening, SSI, prosthetic joint infection, DVT, hospital LOS, total blood loss, degree of coronal mechanical axis, coronal outliers, femoral notches, 3-day and 2-year Knee Society Score, and 3-day and 2-year range of motion. | Worse coronal alignment, more outliers, and more postoperative vomiting in after-hours group. (UV) | 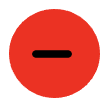 |
| Turrentine, 2010.^133^ | 10426 patients | Non-emergent elective surgery | 30-day mortality, occurrence wound, respiratory, renal, central nervous system, cardiovascular and other complications (further specified), hospital LOS, intraoperative blood transfusion, and return to OR. | Longer LOS and more likeliness to return to OR in nighttime group. (UV) | 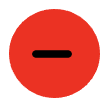 |
| Vimalesvaran, 2013.^134^ | 106 patients | Balloon atrial septostomy | 30-day mortality, mortality immediately after procedure, late mortality after procedure, total mortality, total overall, neurological, cardiac, thrombus, bleeding and miscellaneous complications (further specified), immediate neurological problems after procedure, neurological problems after surgery, arrhythmia, heart failure/anaemia, atrial thrombosis, pericardial effusion, acute pericardial tamponade with effusion, atrioventricular block, atrial flutter, tachycardia, thrombus immediately after procedure, thrombus after surgery, excessive bleeding from groin, clot compressing left atrium posteriorly, necrotizing enterocolitis, chylothorax, acidosis, saturations dropped after procedure, pneumothorax, acute ischaemic lesion, catheter accidentally inserted into right femoral artery, intubation days after procedure, and postoperative arterial oxygen saturations. | Higher total complication rate, higher rate thrombus immediately after balloon atrial septostomy, higher 30-day mortality and higher overall mortality rate in out-of-hours group compared to routine hours group. (UV) | 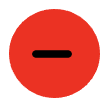 |
| Vinden, 2013.^135^ | 10,155 patients | Laparoscopic cholecystectomy | 30-day mortality, 30-day iatrogenic injuries, and conversion to open surgery. | No significant difference in surgical outcome. | NA |
| Wan, 2021.^136^ | 497 patients | Distal pancreatectomy | 90-day mortality, occurrence any, any > Clavien-Dindo grade II, any > Clavien-Dindo grade III postoperative complication (further specified), occurrence cardiac or pulmonary complication (not further specified), clinically relevant pancreatic fistula, post-pancreatectomy haemorrhage, delayed gastric emptying, abdominal and wound infection, thromboembolic event, renal insufficiency, urinary retention, UTI, total unnecessary damage to adjacent vessels and organs, adjacent organ injury, adjacent vascular injury, vascular injury (grade II), hospital LOS, estimated blood loss, blood transfusion, postoperative percutaneous drainage, reoperation, readmission. | Lower estimated blood loss and higher adjacent organ injury if surgeon worked >5 hours prior to surgery. (UV) | 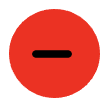/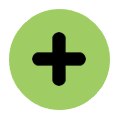 |
| Wang, 2020.^137^ | 117 patients | Radical gastrectomy | Overall survival, occurrence postoperative complications (further specified), total intraoperative organ injury, postoperative LOS, estimated blood loss, and time of resuming oral intake. | Higher blood loss and longer time before resuming oral intake in afternoon group compared to morning group. Lower overall survival distal gastrectomy group when surgery started after 13:00. (UV) | 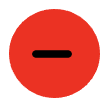 |
| Wendling‐Keim, 2022.^138^ | 387 patients | Osteosynthesis of diverse fractures of extremities in children | Occurrence complications (further specified). | No significant difference in surgical outcome. | NA |
| Wu, 2014.^139^ | 1140 patients | Laparoscopic cholecystectomy | 30-day mortality, occurrence perioperative complications within 30 days of hospitalization (further specified), overall hospital and postoperative LOS, and conversion rate to open surgery. | Higher conversion rate to open surgery in nighttime group compared to daytime group. (MV) | 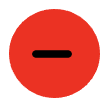 |
| Yaghoubian, 2010.^140^ | 1432 patients | Urgent/emergent trauma surgery | Mortality, median probability of survival, total complications (further specified), cardiac arrest, disseminated intravascular coagulation, empyema, evisceration, coagulopathy, liver failure, PE, pulmonary insufficiency, DVT, wound infection, renal failure, sepsis, pneumonia, and LOS. | More pulmonary insufficiency, less renal failure, and less pneumonia in nighttime group. (UV) No difference in overall complications in multivariate analysis. | 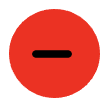/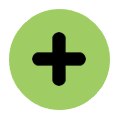 |
| Yang, 2021.^141^ | 740 patients | Adult lung transplant recipients undergoing transplantation surgery | 5-year overall and bronchiolitis obliterans syndrome (BOS)-free survival, occurrence any major postoperative complications within 30 days of transplantation or during entire hospitalization if not discharged within 30 days (further specified), occurrence airway complication (further specified), delayed chest closure, pneumonia, bronchopleural fistula, PE, tracheostomy, reintubation, grade 3 primary graft dysfunction within 72 hours postoperatively, and unexpected return to OR. | Higher risk of any major postoperative complications, 5-year survival, and 5-year BOS-free survival in nighttime group compared to daytime group. (MV) Increase delayed chest closure, pneumonia, and less airway complications in nighttime group. (UV) | 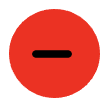/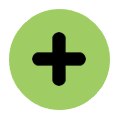 |
| Yount, 2015.^142^ | 3395 patients | Non-emergent cardiac operations | 30-day mortality, total major complications (further specified), deep sternal wound infection, prolonged ventilation, pneumonia, sepsis, renal failure, dialysis, reoperation, stroke, MI, cardiac arrest, atrial fibrillation, postoperative and ICU LOS, ICU readmission, and intraoperative blood transfusion. | Higher mortality rate for patients undergoing late operations compared with cases starting earlier. (MV) Higher rate of deep sternal wound infection in late operation cohort. (UV) | 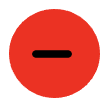 |
| Van Zaane, 2015.^143^ | 11290 patients | Emergency surgery (orthopaedic, breast, gynaecology, vascular, GI, hepatobiliary, plastic, urology, kidney, head/neck, other) | In-hospital mortality, hospital LOS, and no. ICU admissions. | No significant difference in surgical outcome. ^(F)^ | NA |
| Zafar, 2015.^144^ | 31205 patients | Exploratory laparotomy surgery | In-hospital mortality. | No significant difference in surgical outcome. | NA |

(N)ICU = (neonatal) intensive care unit; LOS = length of stay; TIA = transient ischemic attack; CVA = cerebrovascular accident; CABG = coronary artery bypass graft; MI = myocardial infarction; PE= pulmonary embolism; DVT = deep vein thrombosis; UTI = urinary tract infection; GI = gastro-intestinal; SSI = surgical site infection; PSA = prostate-specific antigen; PCA = patient-controlled analgesia; OR = operating room; RBCs = Red blood cells; Complication = specific complications mentioned; Occurrence complications (not) further specified) = increase/decrease in general complication occurrence when fatigued (yes/no), (not) further specified what complications specifically consisted of.

1. Nerve sparing was lower in the afternoon compared to the morning. However, according to the authors, this was likely explained by the larger proportion of Gleason 8/9 tumors in the afternoon cohort. Therefore, it was not mentioned as clinically meaningful.
2. NICU admissions were increased at night, but as cord blood pH and base excess were not significantly worse on the night shift, the authors stated that it might be more the neonatal assessment and not the obstetric treatment that differs at night. Therefore, it was not mentioned as clinically meaningful.
3. There was lower PCA morphine usage in afterhours surgery group, but this could be attributed to the more likelihood of patients having less pian while sleeping throughout the night, as compared to surgery performed during daytime. The authors therefore concluded that after-hour surgery had similar safety and outcome compared to daytime surgery.
4. According to the authors, the absolute difference observed is small and likely not clinically relevant, but as airway dehiscence is a rare complication that may be influenced by surgeon fatigue, it does require further evaluation. Furthermore, there was a small significant difference in 90-day mortality, but according to the authors, it was speculated that this difference is detectable due to the high power associated with the large registry data sets and is therefore not clinically meaningful.
5. In stratified adjusted analysis, there was a slightly greater protective effect of operating in the evening versus the day in perforated appendicitis. However, as the point estimates overlap with the confidence interval, no modification was noted by the authors.
6. The authors reported that patients who underwent emergency or urgent surgery were more often admitted to the ICU during the night. An explanation for this was that either these patients were sicker, or that this was due to logistical issues, as there is no alternative to ICU admission because a suitable alternative care facility was unavailable. Therefore, it was not mentioned as clinically meaningful.

**Supplementary Table 5: Outcome measures included in real-life studies.**

| **Category** | **Subcategory 1** | **Subcategory 2** | **Specifically mentioned** |
| --- | --- | --- | --- |
| Patient mortality  and survival  (N = 113) | Mortality (n = 83) | Overall mortality (n = 7) | - Overall mortality (n = 7) (*1 NEG.*) |
|  |  | Perioperative mortality (n = 1) | - Perioperative mortality (n =1) |
|  |  | Intraoperative mortality (n = 1) | - Intraoperative mortality (n = 1) |
|  |  | Postoperative mortality (n = 2) | - Postoperative mortality (n = 2) (*1 NEG.*) |
|  |  | In-hospital mortality (n = 20) | - In-hospital mortality (n = 20) (*3 NEG.*) |
|  |  | Time-specific mortality (n = 41) | - Mortality immediately after procedure (n = 1) - Death within 24 hours (n = 1) - 7-day mortality (n = 1) (*1 NEG.*) - 30-day mortality (n = 27) (*4 NEG.*) - 90-day mortality (n = 4) (*1 NEG.)* - Mortality >6 months after surgery (n = 1) - One-year mortality (n = 3) - Two-year mortality (n = 1) - 5-year mortality (n = 2) |
|  |  | Other mortality measures (n = 11) | - In-hospital or within 30 days (n = 4) - Maternal death (n =1) - Infant death (n =1) - Cancer specific mortality (n = 1) - Death before cardiopulmonary bypass (n = 1) - Death after major complications (n = 1) - Death <30 days due to any cause or >30 days due to procedure related complication (n = 1) - Total mortality (immediate, 30-day and late) (n = 1) *(1 NEG.)* |
|  | Survival (n = 30) | Overall survival (n = 7) | - Overall survival (n = 7) (*2 NEG.*) *(1 POS.)* |
|  |  | Time-specific survival (n = 19) | - 7-day survival (n = 1) - 30-day survival (n = 4) - 90-day survival (n = 3) - One-year survival (n = 6) (*1 NEG.*) - Two-year survival (n = 1) - Three-year survival (n = 1) (*1 NEG.*) - Five-year survival (n = 3) (*1 NEG.*) |
|  |  | Disease-free survival (n = 3) | - 5-year bronchiolitis obliterans syndrome-free survival (n = 1) (*1 NEG.*) - 5-year disease free survival (n = 1) - Disease free survival (n = 1) (*1 NEG.)* |
|  |  | Other (n = 1) | - Median probability of survival (n = 1) |
| Graft- and transplant-failure and survival  (N = 58) | Graft failure (n = 20) | Overall graft failure (n = 3) | - Overall graft failure (n = 1) (*1 NEG.*) - Graft/prosthetic failure (n = 2) *(1 NEG.)* |
|  |  | Time-specific graft failure (n = 3) | - 30-day graft failure (n = 1) - One-year graft failure (n = 1) - Three-year graft failure (n = 1) |
|  |  | Acute rejections (n = 8) | - Acute rejections (n = 5) - Biopsy proven acute rejections (n = 2) - Clinically inferred acute rejections (n = 1) |
|  |  | Other graft failure measures (n = 6) | - Pure-technical graft failure (n = 1) (*1 POS.*) - Technical graft failure due to vascular problems (n = 1) (*1 POS.*) - Technical graft failure due to urological problems (n = 1) - Technical graft failure due to other problems (n = 1) (*1 POS.*) - Nonimmunological graft failure (n = 1) - Non-viable kidney (n = 1) |
|  | Graft survival (n = 20) | Overall graft survival (n = 5) | - Overall (long-term) graft survival (n = 5) |
|  |  | Time-specific graft survival (n = 13) | - 7-day graft survival (n =1) - 30-day graft survival (n = 3) - 90-day graft survival (n = 3) - One-year graft survival (n = 4) (*1 NEG.*) - Three-year graft survival (n = 1) - Five-year graft survival (n = 1) |
|  |  | Death-censored graft survival (n = 2) | - Death-censored graft survival (n = 2) |
|  | Graft function (n = 15) | Overall graft function (n = 2) | - Overall graft function (n = 2) |
|  |  | Primary non-function (n = 4) | - Primary non-function (n = 4) |
|  |  | Delayed graft function (n = 6) | - Delayed graft function (n = 6) |
|  |  | Slow graft function (n = 1) | - Slow graft-function (n = 1) |
|  |  | Other graft function (n = 2) | - Time before post graft diuresis (n = 1) - Grade 3 primary graft dysfunction <72 hours postoperative (n = 1) |
|  | Transplant failure (n = 3) | Time-specific transplant failure (n =3) | - 30-day transplant failure (n = 1) - One-year transplant failure (n = 1) - Three-year transplant failure (n = 1) |
| Complication occurrence  (N = 167) | Occurrence complications (yes/no) (n = 140) | Not further specified (n = 47) ^(A)^ | - Occurrence complications (n = 7) *(1 NEG.)* - Occurrence postoperative complications (n = 4) - Occurrence intraoperative complications (n = 3) (*1 NEG.*) - Occurrence in-hospital complications (n = 2) - Occurrence general, intraoperative, or postoperative complications (n = 1) - Occurrence surgical complications (n = 2) - Occurrence medical complications (n = 1) - Occurrence serious/major complications (n = 2) - Occurrence vascular complications (n = 2) - Occurrence cardiovascular system complications (n = 1) - Occurrence cardiac complications (n = 1) - Occurrence pulmonary complications (n = 1) - Occurrence biliary complications (n = 1) - Occurrence urologic complications (n = 1) - Occurrence postoperative neurological deficits (n = 1) - Occurrence postoperative neurovascular complications (n = 1) - Occurrence wound complications (n = 1) - Occurrence high-grade complications (n = 1) - Occurrence classification of complications (n = 1) - Occurrence Clavien Dindo complications (I – II, III – IV, or separate grade (n = 9) - Occurrence other complications (n = 4) |
|  |  | Further specified (n = 93) ^(A)^ | - Any complication (n = 11) (*2 NEG.*) - Any major complication (n = 2) *(2 NEG.)* - Any minor complications (n = 1) - Any maternal complications (n = 1) *(1 NEG.)* - Any neonatal complication (n = 1) - Occurrence complications (n = 6) (*1 NEG.*) - Occurrence intraoperative complications (n = 4) (*1 NEG.*) - Occurrence postoperative complications (n = 5) (*1 NEG.*) - Occurrence operative complications (n = 2) - Occurrence perioperative complications (n = 1) - Occurrence medical complications (n = 1) - Occurrence surgical complications (n = 4) - Occurrence major (surgical) complications (n = 4) - Occurrence minor surgical complications (n = 2) - Occurrence long-term complications (n = 1) - Occurrence complications within 30 days (n = 1) - Occurrence systemic perioperative complication (n = 1) - Occurrence (postoperative) cardiac complication (n = 2) - Occurrence cardiovascular complications (n = 1) - Occurrence cardiopulmonary complications (n = 1) - Occurrence respiratory/pulmonary complications (n = 5) (*1 NEG.) (1 POS.)* - Occurrence GI complications (n = 2) - Occurrence biliary complications (n = 2) - Occurrence renal/urologic complications (n = 5) - Occurrence neurologic complications (n = 4) - Occurrence CNS complications (n = 1) - Occurrence vascular complications (n = 4) - Occurrence bleeding complications (n = 3) - Occurrence wound complications (n = 5) - Occurrence infectious complications (n = 3) - Occurrence arthroplasty perioperative complications (n = 1) (*1 NEG.)* - Occurrence organ/visceral injury related complications (n = 1) - Occurrence complications requiring reoperation (n = 1) - Occurrence Clavien Dindo complications (n = 1) - Occurrence other complications (n = 3) |
|  | Total number of complications (n = 27) | Total number of complications (n = 27) | - Total no. of complications (n = 10) (*4 NEG.*) - Total no. of intraoperative complications (n = 1) (*1 NEG.*) - Total no. of postoperative complications (n = 4) (*3 NEG.*) - Total no. of perioperative complications (n = 1) - Total no. of minor complications (n = 2) - Total no. of major complications (n = 2) - Total no. of surgical complications (n = 1) - Total no. of cardiac complications (n = 1) - Total no. of GI complications (n = 1) - Total no. of neurological complications (n = 1) - Total no. of thrombus in femoral vein (n = 1) - Total no. of bleeding complications (n = 1) - Total no. of miscellaneous complications (n = 1) |
| Specific complications  (N = 416) | System-based complications (n = 183) | Cardiac complications (n = 45) | - Myocardial infarction (MI) (n = 11) (*1 NEG.*) - Cardiac arrest (n = 8) (*2 NEG.*) - Atrial fibrillation (n = 5) - Cardiac event (n = 2) - Cardiac tamponade (n = 2) - Myocardial ischemia (n = 1) - Cardiac arrest or pacemaker (n = 1) - Dysrhythmia (n = 1) (1 *NEG.*) - Adverse EKG changes (n = 1) - Conduction block (n = 1) - Hypothermic circulatory arrest (n = 1) - Neonatal arrhythmia (n = 1) - Neonatal heart failure and anaemia (n = 1) - Neonatal atrial thrombus due to balloon rupture (n = 1) - Neonatal pericardial effusion (n = 1) - Neonatal acute pericardial tamponade with effusion (n = 1) - Neonatal atrial flutter (n = 1) - Neonatal atrioventricular block (n = 1) - Neonatal tachycardia (n = 1) - Clot compressing left atrium (n = 1) - Low output syndrome (n = 1) - Last epinephrine infusion dose (representing cardiac output state) (n = 1) |
|  |  | Pulmonary complications (n = 46) | - Pulmonary embolism (n = 9) (*1 NEG.) (1 POS.)* - Failure to wean of ventilation for >48 hours (n = 6) (*4 NEG.*) - Prolonged ventilation (n = 2) - Prolonged ventilation >24 hours (n = 2) - Postoperative need for ventilation (n = 1) - No. ventilator days (n = 1) - Unplanned intubation (n = 3) (*3 NEG.*) - Reintubation after initial intubation (n = 4) (*1 NEG.) (1 POS.)* - Length of initial intubation (n = 1) - Neonatal intubation days after procedure (n = 1) - Not extubated in OR (n = 1) - Pulmonary insufficiency (n = 2) *(1 NEG.)* - Respiratory failure (n = 1) - Airway dehiscence (n = 1) (*1 NEG.*) - Pneumothorax (n = 2) - Hemothorax (n = 1) - Hydrothorax (n = 1) - Chylothorax (n = 3) - Empyema (n = 1) - Saturations dropped after procedure (n = 1) - Prolonged air leak for >5 days (n = 1) - Bronchopleural fistula (n = 1) |
|  |  | Gastrointestinal (GI) complications (n = 15) | - Ileus (n = 5) - Liver failure (n = 2) - Hepatic hypofunction (n = 1) - Biliary fistula (n = 1) - Bile leak / biloma (n = 1) - Bile leak / bile stenosis (n = 1) - Delayed gastric emptying (n = 1) - Mesenteric ischemia / infarction (n = 1) - Flatulence relief time (n = 1) - Postoperative vomiting (n = 1) *(1 NEG.)* |
|  |  | Renal/urologic complications (n = 23) | - Renal failure (n = 10) (*2 NEG.*) *(1 POS.)* - Renal insufficiency (n = 4) - Acute kidney injury (n = 3) - Renal dysfunction (n = 1) - Urinary leak (n = 1) - Urinary necrosis (n = 1) - Urethral stricture (n = 1) - Bladder outflow obstruction/blood clot retention (n = 1) - Urinary retention (n = 1) |
|  |  | Neurologic complications (n = 33) | - Stroke (n = 8) *(1 NEG.)* - Permanent stroke (n = 1) - Cerebral vascular accident (CVA) (n = 3) - Coma (n = 3) - Cerebral infarction (n = 2) - CVA / stroke (n = 2) - Transient ischemic attack (n = 1) - Transient ischemic attach / stroke (n = 1) (1 NEG.) - Delirium / stroke (n = 1) - Spinal cord ischemia (n = 1) - Neonatal seizure (n = 1) - Cerebral vasospasm (n = 1) - Postoperative paralysis (n = 1) - Paraplegia (n = 1) - Psychonosema (n = 1) - CNS events (n = 1) - Neonatal immediate neurological problems after procedure (n = 1) - Neonatal neurological problems after surgery (n = 1) - Hypoxic ischemic insult (n = 1) - Postoperative delirium (n = 1) |
|  |  | Vascular complications (n = 13) | - Renal artery stenosis (n = 1) - Renal vein stenosis (n = 1) - Iliac artery thrombus (n = 1) - Rema; artery aneurysm (n = 1) - Renal pole infarct (n = 1) - Coeliac trunk stenosis (n = 1) - Thrombus immediately after procedure (n = 1) (*1 NEG.*) - Thrombus after surgery (n = 1) - Thromboembolic event (n = 1) - Venous obstruction (n = 1) - Disseminated intravascular coagulation (n = 1) - Hepatic artery thrombosis (n = 1) - Peripheral embolus (n = 1) (*1 NEG.)* |
|  |  | Musculoskeletal complications (n = 4) | - Limb-ischemia (n = 2) - Avascular necrosis femur (n = 1) - Anterior knee pain (n = 1) |
|  |  | Systemic complications (n = 3) | - Hypotension (n = 1) - Multisystem organ failure (n = 2) |
|  |  | Ear-Nose-Throat complications (n = 1) | - Hoarseness (n = 1) |
|  | Infection-related complications (n = 94) | Overall infection (n = 11) | - Infection (n = 5) (*1 NEG.)* - Deep infection (n = 2) - Superficial infection (n = 1) - Drug-treated infection (n = 1) - Infection requiring antibiotics (n = 1) (*1 NEG.*) - Nosocomial infection (n = 1) |
|  |  | Sepsis (n = 11) | - Sepsis (n = 10) (*2 NEG.*) - Septicaemia (n = 1) *(1 NEG.)* |
|  |  | Wound infection (n = 18) | - Wound infection (n = 10) - Deep wound infection (n = 2) (*1 NEG.*) - Deep sternal wound infection (n = 4) (*1 NEG.*) - Superficial wound infection (n = 2) (*1 NEG.*) |
|  |  | Surgical site infection (SSI) / organ space infection (n = 18) | - SSI (n = 7) - Superficial SSI (n = 3) - Deep SSI (n = 1) - Superficial/deep SSI (n = 1) - Organ/space SSI (n = 3) - Organ space infection (n = 1) - SSI location (n = 1) - SSI infection treated by antibiotics, interventional-radiological drainage and antibiotics, or surgical drainage and antibiotics (n = 1) |
|  |  | Cardiovascular infection (n = 1) | - Mediastinitis (n = 1) |
|  |  | Pulmonary infection (n = 16) | - Pneumonia (n = 15) (*3 NEG.*) *(1 POS.)* - Lung infection (n = 1) |
|  |  | GI infection (n = 5) | - Enteritis (n = 1) - (Intra-)abdominal infection (n = 2) - Necrotizing enterocolitis (n = 1) - Abdominal/pelvic abscess (n = 1) |
|  |  | Renal infection (n = 7) | - Urinary tract infection (n = 7) (*2 NEG.*) (*1 POS.)* |
|  |  | Gynaecologic infection (n = 2) | - Postpartum endometritis (n = 2) (*1 NEG.*) |
|  |  | Other infections (n = 5) | - Abscess (n = 3) (*1 POS.*) - (Deep) prosthetic infection (n = 2) |
|  | Wound-related  complications (n = 14) | Wound complications (n =14) | - Wound dehiscence (n = 8) (*2 NEG.*) - Seroma formation (n = 3) - Lymphocele (n = 1) - Impaired wound healing (n = 1) - Wound problem (n = 1) (*1 NEG.)* |
|  | Bleeding complications  (n = 90) | Blood transfusion (n = 29) | - Blood transfusion (n = 21) (*3 NEG.*) - Packed RBCs (n = 2) - Bleeding requiring >4 units RBCs (n = 2) *(1 NEG.)* - Bleeding requiring >3 units RBCs (n = 1) - One-unit packed RBC recruitment (n = 1) - Fresh frozen plasma (n = 2) |
|  |  | Presence bleeding (n = 19) | - Haemorrhage / bleeding (n = 12) (*1 NEG.)* - Hematoma (n = 7) (*1 NEG.*) |
|  |  | Blood loss (n = 30) | - Estimated blood loss (n = 25) (*3 NEG.*) (*1 POS.*) - Shift/postoperative haemoglobin (n = 3) - Shift/postoperative haematocrit (n = 2) |
|  |  | Reoperation for bleeding (n = 12) | - Reoperation for bleeding (n = 12) *(1 NEG.)* |
|  | Procedural complications  (n = 18) | Anastomotic complications (n = 8) | - Anastomotic leak (n = 4) - Anastomotic dehiscence (n = 1) - Anastomotic stenosis (n = 1) - Anastomotic leak or fistula (n = 1) - Anastomotic leak or abscess (n = 1) |
|  |  | Flap-related complications (n = 3) | - Threatened flap (n = 1) - Total flap loss (n = 1) (*1 NEG.*) - Partial flap loss (n = 1) |
|  |  | Stent complications (n = 3) | - In-hospital stent thrombosis (n = 1) - 30-day stent thrombosis (n = 1) - Urinary stent complications (n = 1) |
|  |  | Other procedural complications (n = 4) | - Pancreatic fistula (n = 1) - Lead dislodgement (n = 1) (*1 NEG.*) - Set-screw problem (n = 1) - Aseptic loosening (n = 1) |
|  | Deterioration initial problem (n = 2) | Deterioration initial problem (n = 2) | - Extension dissection TAAAD (n = 1) - Intraoperative rupture aneurysm (n = 1) |
|  | Other complications (n = 15) | Other complications (n = 15) | - Deep vein thrombosis (DVT) (n = 6) (*1 NEG.*) - DVT/thrombophlebitis (n = 2) (*2 NEG.*) - Postoperative malperfusion syndrome (n = 1) - Drug reaction (n = 1) - Peritoneal effusion (n = 1) - Painful hardware (n = 1) - Acidosis (n = 1) - Coagulopathy (n = 1) - Time before resuming oral intake (n = 1) (*1 NEG.*) |
| Iatrogenic event  (N = 51) | Induced injury/deformity  (n = 45) | Overall injury/deformity (n = 5) | - Total organ/vascular damage (n = 1) - Occurrence procurement related organ or vessel injury (n = 1) (*1 NEG.*) - Rate of induced deformity (n = 1) - Injury caused by medical management (n = 1) - 30-day iatrogenic injuries (n = 1) |
|  |  | Organ injury (n = 15) | - Adjacent (intraoperative) organ injury (n = 3) (*2 NEG.*) - Bowel injury (n = 3) - Bladder injury (n = 1) - Cystotomy (n = 1) - Urethral injury (n = 1) - Cardiac perforation (n = 1) - Valve injury (n = 1) - Common bile duct injury (n = 1) - Ureteral perforations (n = 1) - Ureteral avulsions (n = 1) - Perioperative myocardial injury (n = 1) |
|  |  | Nerve injury (n = 8) | - (Peripheral) nerve injury (n = 4) - Intraoperative nerve root injury (n = 1) - Neonatal brachial plexus injury (n = 1) - Traumatic facial nerve palsy (n = 1) - No. nerve sparing (n = 1) |
|  |  | Vascular injury (n = 5) | - Vascular injury (n = 2) - Adjacent vascular injury Grade II (n = 1) - Arterial injury (n = 1) - Coronary venous dissection (n = 1) |
|  |  | Fracture (n = 8) | - Neonatal clavicular fracture (n = 2) - Other neonatal fractures (n = 2) - Neonatal skull fracture (n = 1) - Periprosthetic fracture (n = 1) - Intraoperative fracture (n = 1) - Additional orthopaedic damage (n = 1) |
|  |  | Other injuries/deformities (n = 4) | - Perforation (n = 1) - Fetal lacerations (n = 1) - Incidental dural tear (n = 1) (*1 NEG.*) - Catheter inserted into wrong artery (n = 1) |
|  | Aberrant surgical technique (n = 6) | Aberrant surgical technique (n = 6) | - Fascia closed after laparotomy (n = 1) - Ostomy created after colon resection (n = 1) - Evisceration (n = 1) - Creation of enterostomy (n = 1) - Abdomen left open (n = 1) - Delayed chest closure (n = 1) *(1 NEG.)* |
| Length of stay  (LOS)  (N = 81) | LOS (n = 81) | Overall LOS (n = 12) | - LOS (n = 8) *(1 NEG.) (1 POS.)* - Total LOS (n = 2) (*1 POS.*) - Mean LOS (n = 1) - Initial LOS (n = 1) |
|  |  | Hospital LOS (n = 40) | - In-hospital LOS (n = 40) (*10 NEG.)* (*5 POS.)* |
|  |  | ICU LOS (n = 16) | - Intensive care unit (ICU) LOS (n = 16) *(1 NEG.) (1 POS.)* |
|  |  | Postoperative LOS (n = 12) | - Postoperative LOS (n = 12) *(1 NEG.)* (*2 POS.)* |
|  |  | Prolonged hospitalization >7 days (n = 1) | - Prolonged hospitalization > 7 days (n = 1) (*1 NEG.)* |
| Recurrent or additional problems  (N = 95) | Recurring problems (n = 7) | Recurrent MI (n = 2) | - In-hospital recurrent MI (n = 1) - 30-day recurrent MI (n = 1) |
|  |  | Cancer recurrence (n = 2) | - Local cancer recurrence (n = 1) - Systemic cancer recurrence (n = 1) |
|  |  | PSA recurrence (n = 2) | - PSA recurrence (n = 2) |
|  |  | Refracture (n = 1) | - Refracture (n = 1) |
|  | Readmission (n =18) | Readmission (n = 18) | - Readmission (n = 5) - 30-day readmission (n = 5) - One-year readmission (n = 1) - Five-year readmission (n = 1) - Length of readmission (n = 1) - Maternal readmission (n = 1) - Intensive care unit (ICU) readmission (n = 1) - Readmission due to (late) complications (n = 2) - Readmission related to 30-day complication (n = 1) |
|  | (N)ICU admission (n = 5) | ICU admission (n = 3) | - Intensive care unit (ICU) admission (n = 3) |
|  |  | NICU admission (n = 2) | - Neonatal intensive care unit (NICU) admission (n = 2) |
|  | Need for repeated surgery  (n = 27) | (Need for) reoperation (n = 23) | - Reoperation (n = 10) (*2 NEG.)* - Re-transplantation (n = 1) - Relaparotomy (n = 1) - Cardiac reoperation (n = 1) - Repeat thoracotomy (n = 1) - Reoperation related to aorta (n = 1) - Need for repeat surgery due to technical error (n = 1) - Transplant-related reoperation (n = 1) - Urethral stenosis/leakage requiring reoperation (n = 1) - Vascular stenosis/leakage requiring reoperation (n = 1) (*1 NEG.*) - Graft vein thrombosis requiring reoperation (n = 1) - Re-exploration/revision (n = 3) (*1 NEG.*) |
|  |  | Unplanned return to the OR (n = 4) | - Unplanned return to operating room (OR) (n = 4) (*1 NEG.*) |
|  | Need for additional surgery / interventions (n = 25) | Additional surgical procedures (n = 13) | - Tracheotomy (n = 2) - Pacemaker placement (n = 2) - Tracheostomy (n = 1) - Caesarean hysterectomy (n = 1) - Coronary artery bypass graft (n = 1) - Need for urgent cardiac surgery (n = 1) - Need for other (noncardiac) surgery (n = 1) - Nephrectomy for ischemia (n = 1) - Removal of painful hardware (n = 1) (*1 NEG.*) - Premature removal of tissue expander (n = 1) *(1 NEG.)* - No. multiple access (n = 1) |
|  |  | Additional interventions (n = 12) | - Postoperative dialysis (n = 4) - Endoscopic retrograde cholangiopancreatography (n = 1) - Postoperative intra-aortic balloon pump (n = 1) - Postoperative hemofiltration (n = 1) - Extracorporeal circulatory membrane oxygenation (n = 1) - Continuous renal replacement therapy (n = 2) (*1 NEG.*) - Postoperative percutaneous drainage (n = 1) - Drain placement (n = 1) |
|  | Need for extended surgery  (n = 10) | Conversion to open surgery (n = 10) | - Conversion to open surgery (n = 10) (*1 NEG.*) *(1 POS.)* |
|  | Discharge disposition (n = 3) | Discharge disposition (n = 3) | - Rate of discharge to a facility (n = 1) - Discharge disposition (n = 2) |
| Success or  failure rate  (N = 39) | Success rate (n = 10) | Success rate (n = 4) | - Stone free rate (n = 1) - Flap salvage rate (n = 1) (*1 NEG.*) - Complete stone removal (n = 1) (*1 NEG.*) - Procedural success (likelihood to achieve final angiographic TIMI flow 3) (n = 1) |
|  |  | Successful functional outcome (n = 6) | - Postoperative ambulation weight baring status (n =1) - Good lateral + AP reduction (n = 1) - 2-year Range of motion (ROM) (n = 1) - 3-year Range of motion (ROM) (n = 1) - Postoperative continence (n = 1) - Postoperative potency (n = 1) |
|  | Surgical margin status (n = 8) | Margin status (n = 8) | - Positive margin status (n = 5) - Surgical margin status (n = 1) - Median proximal resection margin (n = 1) (1 POS.) - Median distal resection margin (n = 1) |
|  | Unfavourable outcome (n = 16) | Failure rate (n = 2) | - Implant failure (n = 2) |
|  |  | (Partially) unsuccessful procedures (n = 3) | - Partial stone removal n = 1) - Unsuccessful ureteroscopy (n = 1) (*1 NEG.)* - Unsuccessful reduction (n = 1) |
|  |  | Unsuccessful functional outcome (n = 11) | - Rate of functional loss (n = 1) - Unfavourable outcome at discharge (n = 1) - Unfavourable outcome at 6-month follow-up (n = 1) - AP malreduction (n = 1) - AP + lateral malreduction (n = 1) - Lateral malreduction (n = 1) - No. residual fragments (n = 1) - Retained stone (n = 1) - Poor fixation (n = 1) (*1 NEG.*) - Nonunion (n = 1) - Healing complication (defined as nonunion, delayed union or malalignment) (n = 1) |
|  | Other (n = 5) | Pain management (n = 2) | - Total PCA morphine usage (n = 1) - Additional analgesics consumption (Ketorolac and Demerol) (n = 1) *(1 NEG.)* |
|  |  | Need for IV fluid (n = 2) | - Intravenous (IV) fluid injections >2 days (n = 1) - Intraoperative IV fluids (n = 1) |
|  |  | Radiation exposure (n = 1) | - Radiation exposure (n = 1) |
| Laboratory, radiological, or questionnaire results  (N = 42) | Laboratory results (n = 13) | Postoperative lab-results (n = 10) | - Creatinine 1 week post-transplant (n = 1) - Creatinine 4 weeks post-transplant (n = 1) - Creatinine 24 weeks post-transplant (n = 1) - Creatinine 1 year post-transplant (n = 1) - Creatinine 60 weeks post-transplant (n = 1) - Creatinine at last follow-up (n = 1) *(1 POS.)* - Cord pH <7 (n = 2) - Base excess <12 (n = 1) - Peak serum values of alanine transaminase and aspartate transaminase (n = 1) |
|  |  | Intra-operative lab-results (n = 3) | - Intraoperative pH (n = 1) - Intraoperative HCO3- levels (n = 1) - Intraoperative lactate levels (n = 1) |
|  | Radiological results (n = 17) | Radiological results (n = 16) | - Postoperative Cobb angle (n = 1) - Correction rate (n = 1) - Side bending flexibility (n = 1) - Side bending correction index (n = 1) - Postoperative difference femoral version between fixed and uninjured sides (n = 1) *(1 POS.)* - Postoperative difference femoral length between fixed and uninjured sides (n = 1) - Acetabular component inclination angle > 45 degrees (n = 1) - Femoral component valgus/varus angle >3 degrees (n = 1) - Tibial component valgus/varus angle >3 degrees (n = 1) - Lateral capitellohumeral angle on postoperative early radiographs (n = 1) - Baumann angle on postoperative early radiographs (n = 1) - Anterior humeral line on postoperative early radiographs (n = 1) - Tip apex index (adequacy fixation) (n = 1) - Degree of coronal mechanical axis (n = 1) *(1 NEG.)* - Coronal outliers (n = 1) *(1 NEG.)* - Femoral notches (n = 1) |
|  |  | Angiographic results (n = 1) | - Aneurysm occlusion (n = 1) |
|  | Pathological variables (n = 3) |  | - No. Harvested lymph nodes (n = 1) - No. lymphadenectomy (n = 1) - No. stations harvested lymph nodes (n = 1) |
|  | Questionnaire results (n = 7) | Questionnaire scores (n = 7) | - SF-12 physical scores (n = 1) - SF-12 mental scores (n = 1) - WOMAC-scores (n = 1) - Harris Hip Score (n = 1) - Knee injury and osteoarthrosis score (KOOS) (n = 1) - 3-day Knee Society Score (n = 1) - 2-year Knee Society Score (n = 1) |
|  | Other results (n = 2) |  | - 5-min APGAR < 7 (n = 1) - Postoperative arterial oxygen saturation (n = 1) |
| Composite outcome measures  (N = 6) | Composite outcome measure (n = 6) | Composite outcome (n = 6) | - 30-day composite outcome of death, readmission, or complications (n = 1) - 30-day cardiac composite outcome of death, MI, or vessel revascularization (n = 1) (*1 NEG.*) - Composite outcome of ICU, 30-day readmission or death (n = 1) - Composite outcome of in-hospital death, acute postoperative MI, neurologic morbidity, serious infectious morbidity, new-onset renal failure requiring dialysis or postoperative ventilatory support > 72h (n = 1) - Composite outcome of complications requiring reoperation and/or readmission and premature removal of the tissue expander (n = 1) *(1 NEG.)* - Binary composite outcome of in-hospital death or major complication (n = 1) |

Red = Outcome measure negatively affected by fatigue; Orange = outcome measure both negatively and positively affected by fatigue; Green = outcome measure positively affected by fatigue. Behind the red, orange, and green color-coded outcome measures is stated in brackets how many outcome measures specifically are negatively or positively affected by fatigue, e.g. (*1 NEG*.), meaning one outcome measure is negatively affected by fatigue, or (*3 POS*.), meaning three outcome measures are positively affected by fatigue.

GI = gastro-intestinal; CNS = central nervous system; EKG = electrocardiogram; CVA = cerebrovascular accident; MI = myocardial infarction; AP = anteroproximal; PCA = patient-controlled analgesia.

1. The general occurrence was defined as further specified, if the study explained of what specific complications the group consisted of, e.g., further specifying that *neurologic morbidity* consisted of *central nervous system irritability, hypotonia, coma, or convulsions.* Nonetheless, whether the group was further specified or not, it was solely stated whether the group as a whole was affected by fatigue, and thus no statements were made regarding the individual complications.

**References**

1.Janhofer DE, Lakhiani C, Song DH. Addressing Surgeon Fatigue: Current understanding and strategies for mitigation. *Plast Reconstr Surg*. 2019;144(4):693e-699e. doi:10.1097/PRS.0000000000006075

2.Wetzel CM, Kneebone RL, Woloshynowych M, Nestel D, Moorthy K, Kidd J, et al. The effects of stress on surgical performance. *Am J Surg*. 2006;191(1):5-10. doi:10.1016/j.amjsurg.2005.08.034

3.Dawson D, Reid K. Fatigue, alcohol and performance impairment. *Nature.* 1997;388:235. doi:10.1038/40775

4.Head J, Tenan MS, Tweedell AJ, LaFiandra ME, Morelli F, Wilson KM, et al. Prior mental fatigue impairs marksmanship decision performance. *Front Physiol*. 2017;8:680. doi:10.3389/fphys.2017.00680

5.Bray RM, Camlin CS, Fairbank JA, Dunteman GH, Wheeless SC. The effects of stress on job functioning of military men and women. *Armed Forces Soc*. 2001;27(3):397-417. doi:10.1177/0095327X0102700304

6.Armentrout JJ, Holland DA, O’Toole KJ, Ercoline WR. Fatigue and related human factors in the near crash of a large military aircraft. *Aviat Space Environ Med*. 2006;77(9):963-970. Retrieved from: <https://pubmed.ncbi.nlm.nih.gov/16964748/>

7.Gawande AA, Zinner MJ, Studdert DM, Brennan TA. Analysis of errors reported by surgeons at three teaching hospitals. *Surgery*. 2003;133(6):614-621. doi:10.1067/msy.2003.169

8.Ellman PI, Kron IL, Alvis JS, Tache-Leon C, Maxey TS, Reece TB, et al. Acute sleep deprivation in the thoracic surgical resident does not affect operative outcomes. Ann Thorac Surg. juli 2005;80(1):60-64; discussion 64-65. doi:10.1016/j.athoracsur.2005.01.034

9.McCormick F, Kadzielski J, Landrigan CP, Evans B, Herndon JH, Rubash HE. Surgeon fatigue: a prospective analysis of the incidence, risk, and intervals of predicted fatigue-related impairment in residents. *Arch Surg*. 2012;147(5):430-435. doi:10.1001/archsurg.2012.84

10.Sandblom G, Sevonius D, Staël von Holstein C. Impact of operative time and surgeon satisfaction on the long-term outcome of hernia repair. *Hernia*. 2009;13(6):581-583. doi:10.1007/s10029-009-0527-9

11.Kahol K, Leyba MJ, Deka M, Deka V, Mayes S, Smith M, et al. Effect of fatigue on psychomotor and cognitive skills. *Am J Surg*. 2008;195(2):195-204. doi:10.1016/j.amjsurg.2007.10.004.

12. Abou-Setta A, Beaupre L, Jones C, Rashiq S, Hamm M, Sadowski C. Appendix 1, Newcastle-Ottawa scale assessment of cohort studies. In: Pain management interventions for hip fracture [Internet]. Rockville (MD): Agency for healthcare research and quality (US); 2011. Retrieved from: https://www.ncbi.nlm.nih.gov/books/NBK56664/

13.Uchal M, Tjugum J, Martinsen E, Qiu X, Bergamaschi R. The impact of sleep deprivation on product quality and procedure effectiveness in a laparoscopic physical simulator: a randomized controlled trial. *Am J Surg*. 2005;189(6):753-757. doi:10.1016/j.amjsurg.2005.03.021.

14.Schlosser K, Maschuw K, Kupietz E, Weyers P, Schneider R, Rothmund M, et al. Call-associated acute fatigue in surgical residents--subjective perception or objective fact? A cross-sectional observational study to examine the influence of fatigue on surgical performance. *World J Surg*. 2012;36(10):2276-2287. doi:10.1007/s00268-012-1699-5

15.Sahar Y, Wagner M, Barel A, Shoval S. Stress-adaptive training: an adaptive psychomotor training according to stress measured by grip force. *Sensors (Basel).* 2022;22(21):8368. doi:10.3390/s22218368.

16.Brandenberger J, Kahol K, Feinstein AJ, Ashby A, Smith M, Ferrara JJ. Effects of duty hours and time of day on surgery resident proficiency. *Am J Surg*. 2010;200(6):814-8; discussion 818-819. doi: 10.1016/j.amjsurg.2010.06.009

17.Gerdes J, Kahol K, Smith M, Leyba MJ, Ferrara JJ. Jack Barney award: the effect of fatigue on cognitive and psychomotor skills of trauma residents and attending surgeons. *Am J Surg*. 2008;196(6):813-9; discussion 819-820. doi:10.1016/j.amjsurg.2008.07.030

18.Kahol K, Smith M, Brandenberger J, Ashby A, Ferrara JJ. Impact of fatigue on neurophysiologic measures of surgical residents. *J Am Coll Surg*. 2011;213(1):29-34; discussion 34-36. doi:10.1016/j.jamcollsurg.2011.03.028

19.Muffly TM, Espaillat-Rijo LM, Edwards AM, Horton A. Operating room fatigue: is your twentieth surgical knot as strong as your first? *J Surg Educ*. 2012;69(2):215-217. doi:10.1016/j.jsurg.2011.09.005

20.Fox S, Kotelba A. Variational principle of least psychomotor action: modelling effects on action from disturbances in psychomotor work involving human, cyborg, and robot workers. *Entropy (Basel).* 2019;21(6):543. doi:10.3390/e21060543

21.Dwyer CP. An evaluative review of barriers to critical thinking in educational and real-world settings. *J Intell*. 2023;11(6):105. doi:10.3390/jintelligence11060105

22.Hamann A, Carstengerdes N. Assessing the development of mental fatigue during simulated flights with concurrent EEG-fNIRS measurement. *Sci Rep*. 2023;13:4738. doi:10.1038/s41598-023-31264-w

23.Lieberman HR, Bathalon GP, Falco CM, Kramer FM, Morgan CA, Niro P. Severe decrements in cognition function and mood induced by sleep loss, heat, dehydration, and undernutrition during simulated combat. *Biol Psychiatry*. 2005;57(4):422-429. doi:10.1016/j.biopsych.2004.11.014

24.van de Ven HA, Hulsegge G, Zoomer T, de Korte EM, Burdorf A, Oude Hengel KM. The acute effects of working time patterns on fatigue and sleep quality using daily measurements of 6195 observations among 223 shift workers. *Scand J Work Environ Health*. 2021;47(6):446-455. doi:10.5271/sjweh.3964

25.Hockey G. A motivational control theory of cognitive fatigue. In: Ackerman PL. Cognitive Fatigue: Multidisciplinary Perspectives on Current Research and Future Applications. American Psychological Association; 2011. p. 167-187. doi:10.1037/12343-008

26.Herlambang MB, Taatgen NA, Cnossen F. The role of motivation as a factor in mental fatigue. *Hum Factors*. 2019;61(7):1171-1185. doi:10.1177/0018720819828569

27.Boksem MAS, Meijman TF, Lorist MM. Mental fatigue, motivation and action monitoring. *Biol Psychol*. 2006;72(2):123-132. doi:10.1016/j.biopsycho.2005.08.007

28.Barte JCM, Nieuwenhuys A, Geurts SAE, Kompier MAJ. Motivation counteracts fatigue-induced performance decrements in soccer passing performance. *J Sports Sci*. 2019;37(10):1189-1196. doi:10.1080/02640414.2018.1548919

29.Herlambang MB, Cnossen F, Taatgen NA. The effects of intrinsic motivation on mental fatigue. *PLoS One*. 2021;16(1):e0243754. doi:10.1371/journal.pone.0243754

30.Guan S, Xiaerfuding X, Ning L, Lian Y, Jiang Y, Liu J, et al. Effect of job strain on job burnout, mental fatigue and chronic diseases among civil servants in the Xinjiang Uygur autonomous region of China. *Int J Environ Res Public Health*. 2017;14(8):872. doi:10.3390/ijerph14080872

31.Demerouti E, Velduis W, Coombes C, Hunter R. Burnout among pilots: psychosocial factors related to happiness and performance at simulator training. *Ergonomics*. 2019;62(2):233-245. doi:10.1080/00140139.2018.1464667

32.Ghasemloonia A, Maddahi Y, Zareinia K, Lama S, Dort JC, Sutherland GR. Surgical skill assessment using motion quality and smoothness. *J Surg Educ*. 2017;74(2):295-305. doi:10.1016/j.jsurg.2016.10.006

33.Wolf A, Ueda K. Contribution of eye-tracking to study cognitive impairments among clinical populations. *Front Psychol*. 2021;12:590986. doi:10.3389/fpsyg.2021.590986

34. Wingelaar-Jagt YQ, Wingelaar TT, Riedel WJ, Ramaekers JG. Fatigue in aviation: safety risks, preventive strategies and pharmacological interventions. *Front Physiol.* 2021;12:712628. doi:10.3389/fphys.2021.712628

35. Pan T, Wang H, Si H, Li Y, Shang L. Identification of pilots’ fatigue status based on electrocardiogram signals. *Sensors (Basel)*. 2021;21(9):3003. doi:[10.3390/s21093003](https://doi.org/10.3390%2Fs21093003)

36.A-Lai GH, Hu JR, Xu ZJ, Yao P, Zhong X, Wang YC, et al. Whether the start time of elective lung surgery impacts perioperative outcomes and cost? *Front Surg.* 2022;9:922198. doi:10.3389/fsurg.2022.922198

37.Ahlsson A, Wickbom A, Geirsson A, Franco-Cereceda A, Ahmad K, Gunn J, et al. Is there a weekend effect in surgery for type A dissection?: results from the Nordic Consortium for acute type A aortic dissection database. *Ann Thorac Surg.* 2019;108(3):770-776. doi:10.1016/j.athoracsur.2019.03.005

38.Alnajashi SS, Alayed SA, Al-Nasher SM, Adebasi B, Khan MM. Will surgeries performed at night lead to worse outcomes? Findings from a trauma center in Riyadh. *Medicine (Baltimore)*. 2020;99(32):e20273. doi: 10.1097/MD.0000000000020273

39.Araujo RLC, Karkar AM, Allen PJ, Gönen M, Chou JF, Brennan MF, et al. Timing of elective surgery as a perioperative outcome variable: analysis of pancreaticoduodenectomy. *HPB (Oxford)*. 2014;16(3):250-262. doi:10.1111/hpb.12107

40.Arnaoutakis G, Bianco V, Estrera AL, Brinster DR, Ehrlich MP, Peterson MD, et al. Time of day does not influence outcomes in acute type A aortic dissection: results from the IRAD. *J Card Surg.* 2020;35(12):3467-3473. doi:10.1111/jocs.15017.

41.Assali AR, Brosh D, Vaknin-Assa H, Fuchs S, Teplitsky I, Sela O, et al. The impact of circadian variation on outcomes in emergency acute anterior myocardial infarction percutaneous coronary intervention. *Catheter Cardiovasc Interv*. 2006;67(2):221-226. doi:10.1002/ccd.20608

42.Axtell AL, Moonsamy P, Melnitchouk S, Jassar AS, Villavicencio MA, D’Alessandro DA, et al. Starting elective cardiac surgery after 3 pm does not impact patient morbidity, mortality, or hospital costs. *J Thorac Cardiovasc Surg.* 2020;159(6):2314-2321. doi:10.1016/j.jtcvs.2019.06.125

43.Aydoğmuş A, Duymuş TM, Keçeci T, Adiyeke L, Kafadar AB. Comparison of daytime and after-hours surgical treatment of supracondylar humeral fractures in children. *J Pediatr Orthop B*. 2017;26(5):400-404. doi:10.1097/BPB.0000000000000403

44.Bagrodia A, Rachakonda V, Delafuente K, Toombs S, Yeh O, Scales J, et al. Surgeon fatigue: impact of case order on perioperative parameters and patient outcomes. *J Urol.* 2012;188(4):1291-1296. doi:10.1016/j.juro.2012.06.021

45.Bailit JL, Landon MB, Thom E, Rouse DJ, Spong CY, Varner MW, et al. The MFMU Cesarean Registry: impact of time of day on cesarean complications. *Am J Obstet Gynecol.* 2006;195(4):1132-1137. doi:10.1016/j.ajog.2006.06.009.

46.Barinaga G, Wright E, Cagle PJ Jr, Anoushiravani AA, Sayeed Z, Chambers MC, et al. Effect of time of operation on hip fracture outcomes: a retrospective analysis. *J Am Acad Orthop Surg.* 2017;25(1):55-60. doi:10.5435/JAAOS-D-15-00494

47.Becker F, Voß T, Mohr A, Mehdorn AS, Schütte-Nütgen K, Reuter S, et al. Impact of nighttime procedures on outcomes after liver transplantation. *PLoS One.* 2019;14(7):e0220124. doi:10.1371/journal.pone.0220124

48.Bekelis K, Missios S, MacKenzie TA. Outcomes of elective cerebral aneurysm treatment performed by attending neurosurgeons after night work. *Neurosurgery.* 2018;82(3):329-334. doi:10.1093/neuros/nyx174

49.Bianco V, Aranda-Michel E, Serna-Gallegos D, Kilic A, Kaczarowski DJ, Dunn-Lewis C, et al. Cardiac surgery in the afternoon is not associated with increased operative morbidity and mortality. *J Card Surg.* 2021;36(10):3599-3606. doi: 10.1111/jocs.15890

50.de Boer J, van der Bogt K, Putter H, Ooms-de Vries K, Haase-Kromwijk B, Pol R, et al. Surgical quality in organ procurement during day and night: an analysis of quality forms. *BMJ Open.* 2018;8(11):e022182. doi:10.1136/bmjopen-2018-022182

51.Boscà A, Montalvá EM, Maupoey J, Argüelles B, Navío A, Calatayud D, et al. Does surgeon fatigue influence the results of liver transplantation? *Transplant Proc.* 2019;51(1):67-70. doi:10.1016/j.transproceed.2018.03.139

52.Canal C, Lempert M, Birrer DL, Neuhaus V, Turina M. Short-term outcome after appendectomy is related to preoperative delay but not to the time of day of the procedure: a nationwide retrospective cohort study of 9224 patients. *Int J Surg.* 2020;76;16-24. doi:10.1016/j.ijsu.2020.02.001

53.Capello SA, Patel HRH, Joseph JV. Surgical case order does not affect outcomes during robot-assisted radical prostatectomy. *J Robot Surg.* 2008;2(1):25-29. doi:10.1007/s11701-007-0066-2

54. Chacko AT, Ramirez MA, Ramappa AJ, Richardson LC, Appleton PT, Rodriguez EK. Does late night hip surgery affect outcome? *J Trauma.* 2011;71(2):447-453; discussion 453. doi:10.1097/TA.0b013e3182231ad7.

55.Chan YM, Tang N, Chow SK. Surgical outcome of daytime and out-of-hours surgery for elderly patients with hip fracture. *Hong Kong Med J.* 2018;24(1):32-37. doi:10.12809/hkmj165044

56.Chang WC, Lin CC, Chen LM, Wu HDI, Yeh LS, Lin LY. Schedule effect and laparoscopic-assisted vaginal hysterectomy. *Aust N Z J Obstet Gynaecol.* 2008;48(4):433-7. doi:10.1111/j.1479-828X.2008.00859.x

57.Chen C, Zhang X, Gu C, Wang Y, Liu K, Pan X, et al. Surgery performed at night by continuously working surgeons contributes to a higher incidence of intraoperative complications in video-assisted thoracoscopic pulmonary resection: a large monocentric retrospective study. *Eur J Cardiothorac Surg.* 2020;57(3):447-454. doi:10.1093/ejcts/ezz253

58.Chiu CK, Chan CYW, Chandren JR, Ong JY, Loo SF, Hasan MS, et al. After-hours elective spine deformity corrective surgery for patients with adolescent idiopathic scoliosis: is it safe? *J Orthop Surg (Hong Kong).* 2019;27(2):2309499019839023. doi:10.1177/2309499019839023.

59.Chu MWA, Stitt LW, Fox SA, Kiaii B, Quantz M, Guo L, et al. Prospective evaluation of consultant surgeon sleep deprivation and outcomes in more than 4000 consecutive cardiac surgical procedures. *Arch Surg.* 2011;146(9):1080-1085. doi:10.1001/archsurg.2011.121

60.Cook TM, Britton DC, Craft TM, Jones CB, Horrocks M. An audit of hospital mortality after urgent and emergency surgery in the elderly. *Ann R Coll Surg Engl.* 1997;79(5):361-367. Retrieved from: https://pubmed.ncbi.nlm.nih.gov/9326129/

61.Coumbe A, John R, Kuskowski M, Agirbasli M, McFalls EO, Adabag S. Variation of mortality after coronary artery bypass surgery in relation to hour, day and month of the procedure. *BMC Cardiovasc Disord.*  2011;11:63. doi:10.1186/1471-2261-11-63

62.Dalton MK, McDonald E, Bhatia P, Davis KA, Schuster KM. Outcomes of acute care surgical cases performed at night. *Am J Surg.* 2016;212(5):831-836. doi:10.1016/j.amjsurg.2016.02.024

63.Ellman PI, Law MG, Tache-Leon C, Reece TB, Maxey TS, Peeler BB, et al. Sleep deprivation does not affect operative results in cardiac surgery. *Ann Thorac Surg.* 2004;78(3):906-911; discussion 906-11. doi:10.1016/j.athoracsur.2004.04.006.

64.Eskesen TG, Peponis T, Saillant N, King DR, Fagenholz PJ, Velmahos GC, et al. Operating at night does not increase the risk of intraoperative adverse events. *Am J Surg.* 2018;216(1):19-24. doi:10.1016/j.amjsurg.2017.10.026

65.Fechner G, Pezold C, Hauser S, Gerhardt T, Müller SC. Kidney’s nightshift, kidney’s nightmare? Comparison of daylight and nighttime kidney transplantation: impact on complications and graft survival. *Transplant Proc.* 2008;40(5):1341-1344. doi:10.1016/j.transproceed.2008.02.072

66.Fernandes S, Carvalho AF, Rodrigues AJ, Costa P, Sanz M, Goulart A, et al. Day and night surgery: is there any influence in the patient postoperative period of urgent colorectal intervention? *Int J Colorectal Dis.* 2016;31(3):525:533. doi:10.1007/s00384-015-2494-1

67.Gabriel RA, A’Court AM, Schmidt UH, Dutton RP, Urman RD. Time of day is not associated with increased rates of mortality in emergency surgery: an analysis of 49,196 surgical procedures. *J Clin Anesth*. 2018;46:85-90. doi:10.1016/j.jclinane.2018.02.004

68.Gasser S, Stastny L, Kofler M, Zujs V, Krapf C, Semsroth S, et al. Surgery out of office hours for type A aortic dissection: does night-time and weekend surgery worsen outcome? *Interact Cardiovasc Thorac Surg.* 2020;31(6):806-812. doi:10.1093/icvts/ivaa190

69.George TJ, Arnaoutakis GJ, Merlo CA, Kemp CD, Baumgartner WA, Conte JV, et al. Association of operative time of day with outcomes after thoracic organ transplant. *JAMA.* 2011;305(21):2193-2199. doi: 10.1001/jama.2011.726

70.Goertz L, Kabbasch C, Pflaeging M, Pennig L, Laukamp KR, Timmer M, et al. Impact of the weekend effect on outcome after microsurgical clipping of ruptured intracranial aneurysms. *Acta Neurochir (Wien)*. 2021;163(3):783-791. doi: 10.1007/s00701-020-04689-9

71.Govindarajan A, Urbach DR, Kumar M, Li Q, Murray BJ, Juurlink D, et al. Outcomes of daytime procedures performed by attending surgeons after night work. *N Engl J Med.* 2015;373(9):845-853. doi: 10.1056/NEJMsa1415994

72.Guidry CA, Davies SW, Willis RN, Dietch ZC, Shah PM, Sawyer RG. Operative start time does not affect post-operative risk infection. *Surg Infect (Larchmt)*. 2016;17(5):547-551. doi:10.1089/sur.2015.150

73. Guo QH, Liu QL, Hu XJ, Li Y, Zheng J, Xue WJ. Comparison of nighttime and daytime operation on outcomes of kidney transplant with deceased donors: a retrospective analysis. *Chin Med J (Engl)*. 2019;132(4):395-404. doi: 10.1097/CM9.0000000000000056

74.Halldorson JB, Bakthavatsalam R, Reyes JD, Perkins JD. The impact of consecutive operations on survival after liver transplantation. *Liver Transpl*. 2009;15(8):907-914. doi:10.1002/lt.21734

75.Halliday N, Martin K, Collett D, Allen E, Thorburn D. Is liver transplantation 'out-of-hours' non-inferior to 'in-hours' transplantation? A retrospective analysis of the UK transplant registry. *BMJ Open*. 2019;9(2):e024917. doi:10.1136/bmjopen-2018-024917

76.Halvachizadeh S, Teuber H, Cinelli P, Allemann F, Pape HC, Neuhaus V. Does the time of day in orthopedic trauma surgery affect mortality and complication rates? *Patient Saf Surg.* 2019;13:8. doi:10.1186/s13037-019-0186-4

77. Haynes DF, Schwedler M, Dyslin DC, Rice JC, Kerstein MD. Are postoperative complications related to resident sleep deprivation? *South Med J.* 1995;88(3):283-289. doi:10.1097/00007611-199503000-00007

78.Heller JA, Kothari R, Lin HM, Levin MA, Weiner M. Surgery start time does not impact outcome in elective cardiac surgery. *J Cardiothorac Vasc Anesth.* 2017;31(1):32-36. doi:10.1053/j.jvca.2016.08.015

79. Hsu JC, Varosy PD, Parzynski CS, Chaudhry SI, Dewland TA, Curtis JP, et al. Procedure timing as a predictor of inhospital adverse outcomes from implantable cardioverter-defibrillator implantation: Insights from the national cardiovascular data registry. *Am Heart J*. 2015;169(1):45-52. doi:10.1016/j.ahj.2014.10.006

80.Ishiyama Y, Ishida F, Ooae S, Takano Y, Seki J, Shimada S, et al. Surgical starting time in the morning versus the afternoon: propensity score matched analysis of operative outcomes following laparoscopic colectomy for colorectal cancer. *Surg Endosc.* 2019;33(6):1769-1776. doi: 10.1007/s00464-018-6449-9

81.Karagoz B, Keceli O, Cukurlu M, Agir I. Comparison of daytime and after-hours surgical treatment of femoral neck fractures. *Niger J Clin Pract.* 2022;25(11):1846-1852. doi:10.4103/njcp.njcp_285_22

82.Kelz RR, Freeman KM, Hosokawa PW, Asch DA, Spitz FR, Moskowitz M, et al. Time of day is associated with postoperative morbidity: an analysis of the national surgical quality improvement program data. *Ann Surg.* 2008;247(3):544-552. doi:10.1097/SLA.0b013e31815d7434

83.Kelz RR, Tran TT, Hosokawa P, Henderson W, Paulson EC, Spitz F, et al. Time-of-day effects on surgical outcomes in the private sector: a retrospective cohort study. *J Am Coll Surg.* 2009;209(4):434-445.e2. doi:10.1016/j.jamcollsurg.2009.05.022

84.Kienzl-Wagner K, Schneiderbauer S, Bösmüller C, Schneeberger S, Pratschke J, Ollinger R. Nighttime procedures are not associated with adverse outcomes in kidney transplantation. *Transpl Int.* 2013;26(9):879-885. doi: 10.1111/tri.12125

85.Kim WJ, Pyon JK, Mun GH, Bang SI, Jeon BJ, Lee KT. Is elective nighttime operation associated with adverse outcomes? Analysis in immediate tissue expander-based breast reconstruction. *J Patient Saf.* 2022;18(4):261-268. doi:10.1097/PTS.0000000000000963

86.Koltka AK, İlhan M, Ali A, Gök AFK, Sivrikoz N, Yanar TH, et al. Is nighttime laparoscopic general surgery under general anesthesia safe? *Ulus Travma Acil Cerrahi Derg.* 2018;24(1):20-24. doi:10.5505/tjtes.2017.95079

87.Kork F, Spies C, Conrad T, Weiss B, Roenneberg T, Wernecke KD, et al. Associations of postoperative mortality with the time of day, week and year. *Anaesthesia.* 2018;73(6):711-718. doi:10.1111/anae.14228

88.Larsen P, Koelner-Augustson L, Elsoe R, Petruskevicius J, Rasmussen S. The long-term outcome after treatment for patients with tibial fracture treated with intramedullary nailing is not influenced by time of day of surgery and surgeon experience. *Eur J Trauma Emerg Surg*. 2017;43(2):221-226. doi:10.1007/s00068-015-0622-9

89.Lee KT, Mun GH. Is after-hours free-flap surgery associated with adverse outcomes? *J Plast Reconstr Aesthet Surg.* 2013;66(4):460-466. doi:10.1016/j.bjps.2012.12.007

90.Li X, Zhang Q, Dong J, Zhang G, Chai W, Chen J. Impact of surgical case order on peri-operative outcomes for total joint arthroplasty. *Int Orthop*. 2018;42(10):2289-2294. doi: 10.1007/s00264-018-3835-9

91.Lim AH, Lane S, Page R. The effect of surgical timing on the outcome of patients with neck of femur fracture. *Arch Orthop Trauma Surg.* 2015;135(11):1497-1502. doi:10.1007/s00402-015-2303-x

92.Lonze BE, Parsikia A, Feyssa EL, Khanmoradi K, Araya VR, Zaki RF, et al. Operative start times and complications after liver transplantation. *Am J Transplant.* 2010;10(8):1842-1849. doi:10.1111/j.1600-6143.2010.03177.x

93.Lu Q, Shen Y, Zhang J, Ren YF, Dong J, Du ZQ, et al. Operation start times and postoperative morbidity from liver resection: a propensity score matching analysis. *World J Surg.* 2017;41(4):1100-1109. doi:10.1007/s00268-016-3827-0

94.Lu Q, Li QS, Zhang W, Liu K, Li T, Yu JW, et al. Operation start time and long-term outcome of hepatocellular carcinoma after curative hepatic resection. *Ann Surg Treat Res.* 2020;99(1):1-7. doi:10.4174/astr.2020.99.1.1

95.Mehra K, Manikandan R, Dorairajan LN, Sreerag KD, Kumar R. Day and night surgery: comparing outcomes in deceased donor renal transplant with surgeons' perspectives to operate at odd hours - a prospective observational study. *Indian J Transplant*. 2020;14(3):224-229. Doi:10.4103/ijot.ijot_26_20

96.Miyahara J, Ohya J, Kawamura N, Ohtomo N, Kunogi J. Adverse effects of surgeon performance after a night shift on the incidence of perioperative complications in elective thoracolumbar spine surgery. *J Orthop Sci.* 2021;26(6):948-952. doi:10.1016/j.jos.2020.09.019

97.Mönttinen T, Kangaspunta H, Laukkarinen J, Ukkonen M. Nighttime appendectomy is safe and has similar outcomes as daytime appendectomy: a study of 1998 appendectomies. *Scand J Surg.* 2021;110(2):227-232. doi:10.1177/1457496920938605

98.Narayan P, Benedetto U, Caputo M, Guida G, Bryan AJ, Angelini GD. Type A acute aortic dissection repair during night time: is it safe? *Indian J Thorac Cardiovasc Surg.* 2020;36(2):114-118. doi: 10.1007/s12055-019-00851-w

99.Nasseri Y, Oka K, Kasheri E, Cohen J, Ellenhorn J, Cox B, et al. Robotic colorectal procedures: does operative start time impact short-term outcome? *Surg Endosc*. 2022;36(8):5669-5675. doi:10.1007/s00464-022-09086-0

100.Ndegbu CU, Olasehinde O, Sharma A, Arowolo OA, Adisa AO, Alatise OI, et al. Daytime versus night-time emergency abdominal operations: perspective from a low-middle-income country. *World J Surg.* 2019;43(12):2967-2972. doi:10.1007/s00268-019-05160-2

101. Okkaoglu MC, Ozdemir FE, Ozdemir E, Karaduman M, Ates A, Altay M. Is there an optimal timing for surgical treatment of pediatric supracondylar humerus fractures in the first 24 hours? *J Orthop Surg Res*. 2021;16(1):484. doi:10.1186/s13018-021-02638-5.

102.Orman ES, Hayashi PH, Dellon ES, Gerber DA, Barritt 4^th^ AS. Impact of nighttime and weekend liver transplants on graft and patient outcomes. *Liver Transpl.* 2012;18(5):558-565. doi:10.1002/lt.23395

103.Özdemir-van Brunschot DMD, Hoitsma AJ, van der Jagt MFP, d’Ancona FC, Donders RART, van Laarhoven CJHM, et al. Nighttime kidney transplantation is associated with less pure technical graft failure. *World J Urol.* 2016;34(7):955-961. doi: 10.1007/s00345-015-1679-0

104.Patel NM, Yoon RS, Koerner JD, Donegan DJ, Liporace FA. Timing of diaphyseal femur fracture nailing: is the difference night and day? *Injury*. 2014;45(3):546-549. doi:10.1016/j.injury.2013.10.018

105.Patel SV, Groome PA, Merchant SJ, Lajkosz K, Nanji S, Brogly SB. Timing of surgery and the risk of complications in patients with acute appendicitis: A population-level case-crossover study. *J Trauma Acute Care Surg.* 2018;85(2):341-347. doi:10.1097/TA.0000000000001962

106.Patella M, Papagiannopoulos K, Milton R, Chaudhuri N, Kefaloyannis E, Brunelli A. Operating room scheduling is not associated with early outcome following elective anatomic lung resections: a propensity score case-matched analysis. *Eur J Cardiothorac Surg*. 2017;15(4):660-666. doi:10.1093/ejcts/ezw371

107.Peled Y, Melamed N, Chen R, Pardo J, Ben-Shitrit G, Yogev Y. The effect of time of day on outcome of unscheduled cesarean deliveries. *J Matern Fetal Neonatal Med.* 2011;24(8):1051-1054. doi:10.3109/14767058.2010.545913

108.Peskun C, Walmsley D, Waddell J, Schemitsch E. Effect of surgeon fatigue on hip and knee arthroplasty. *Can J Surg.* 2012;55(2):81-86. doi:10.1503/cjs.032910

109.Phatak UR, Chan WM, Lew DF, Escamilla RJ, Ko TC, Wray CJ, et al. Is nighttime the right time? Risk of complications after laparoscopic cholecystectomy at night. *J Am Coll Surg.* 2014;219(4):718-724. doi:10.1016/j.jamcollsurg.2014.05.009

110.Qiu J, Zhang L, Luo X, Gao W, Liu S, Jiang W, et al. Higher mortality in patients undergoing nighttime surgical procedures for acute type A aortic dissection. *Ann Thorac Surg*. 2018;106(4):1164-1170. doi:10.1016/j.athoracsur.2018.04.062

111.Rashid RH, Zubairi AJ, Slote MU, Noordin S. Hip fracture surgery: does time of the day matter? A case-controlled study. *Int J Surg*. 2013;11(9):923-925. doi:10.1016/j.ijsu.2013.07.003

112.Ricci WM, Gallagher B, Brandt A, Schwappach J, Tucker M, Leighton R. Is after-hours orthopaedic surgery associated with adverse outcomes? A prospective comparative study. *J Bone Joint Surg Am.* 2009;91(9):2067-2072. doi:10.2106/JBJS.H.00661

113.Rogers ME, Egberg MD, Sylvester F, Lichtman S, Lin FC. Outcomes of pediatric liver transplant are unaffected by the time or day of surgery. *Pediatr Transplant*. 2020;24(8):e13826. doi: 10.1111/petr.13826

114.Rothschild JM, Keohane CA, Rogers S, Gardner R, Lipsitz SR, Salzberg CA, et al. Risks of complications by attending physicians after performing nighttime procedures. *JAMA*. 2009;302(14):1565-1572. doi: 10.1001/jama.2009.1423

115.Schieman C, MacLean AR, Buie WD, Rudmik LR, Ghali WA, Dixon E. Does surgeon fatigue influence outcomes after anterior resection for rectal cancer? *Am J Surg*. 2008;195(5):684-687; discussion 687-8. doi:10.1016/j.amjsurg.2008.01.009

116.Schuster KM, Hazelton JP, Rattigan D, Nguyen L, Kim D, Spence LH, et al. Can acute care surgeons perform while fatigued? An EAST multicenter study. *J Trauma Acute Care Surg.* 2018;85(3):476-484. doi:10.1097/TA.0000000000001975

117.Seklehner S, Heißler O, Engelhardt PF, Hruby S, Riedl C. Impact of hours worked by a urologist prior to performing ureteroscopy on its safety and efficacy. *Scand J Urol.* 2016;50(1):56-60. doi:10.3109/21681805.2015.1079798

118.Seow YY, Alkari B, Dyer P, Riad H. Cold ischemia time, surgeon, time of day, and surgical complications. *Transplantation.* 2004;77(9):1386-1389. doi:10.1097/01.tp.0000122230.46091.e2

119.Sessler DI, Kurz A, Saager L, Dalton JE. Operation timing and 30-day mortality after elective general surgery. *Anesth Analg.* 2011;113(6):1423-1428. doi:10.1213/ANE.0b013e3182315a6d

120.Shah AA, Al-Zoubi RM, Al-Qudimat AR, Amine Rejeb M, Kumari Ojha L, Abdulzem S, et al. Daytime versus nighttime laparoscopic appendectomy in term of complications and clinical outcomes: A retrospective study of 1001 appendectomies. *Heliyon*. 2022;8(12):e11911. doi:10.1016/j.heliyon.2022.e11911

121.Sharpe JP, Weinberg JA, Magnotti LJ, Nouer SS, Yoo W, Zarzaur BL,et al. Outcomes of operations performed by attending surgeons after overnight trauma shifts. *J Am Coll Surg.* 2013;216(4):791-797; discussion 797-9. doi:10.1016/j.jamcollsurg.2012.12.005

122.Shaw TM, Lonze BE, Feyssa EL, Segev DL, May N, Parsikia A, et al. Operative start times and complications after kidney transplantation. *Clin Transplant.* 2012;26(3):E177-183. doi:10.1111/j.1399-0012.2012.1622.x

123.Siada SS, Schaetzel SS, Chen AK, Hoang HD, Wilder FG, Dirks RC, et al. Day versus night laparoscopic cholecystectomy for acute cholecystitis: A comparison of outcomes and cost. *Am J Surg.* 2017;214(6):1024-1027. doi:10.1016/j.amjsurg.2017.08.027

124.Sugünes N, Bichmann A, Biernath N, Peters R, Budde K, Liefeldt L, et al. Analysis of the effects of day-time vs. night-time surgery on renal transplant patient outcomes. *J Clin Med*. 2019;8(7):1051. doi:10.3390/jcm8071051

125.Sun EC, Mello MM, Vaugn MT, Kheterpal S, Hawn MT, Dimick JB, et al. Assessment of perioperative outcomes among surgeons who operated the night before. *JAMA Intern Med.* 2022;182(7):720-728. doi: 10.1001/jamainternmed.2022.1563

126.Switzer JA, Bennett RE, Wright DM, Vang S, Anderson CP, Vlasak AJ, et al. Surgical time of day does not affect outcome following hip fracture fixation. *Geriatr Orthop Surg Rehabil.* 2013;4(4):109-116. doi:10.1177/2151458513518344

127.Tan PJ, Xu M, Sessler DI, Bashour CA. Operation timing does not affect outcome after coronary artery bypass graft surgery. *Anesthesiology*. 2009;111(4):785-789. doi:10.1097/ALN.0b013e3181b6a50c

128.Tessler MJ, Charland L, Wang NN, Correa JA. The association of time of emergency surgery - day, evening or night - with postoperative 30-day hospital mortality. *Anaesthesia.* 2018;73(11):1368-1371. doi:10.1111/anae.14329

129.Thomas M, Allen MS, Wigle DA, Shen KR, Cassivi SD, Nichols 3^rd^ FC, et al. Does surgeon workload per day affect outcomes after pulmonary lobectomies? *Ann Thorac Surg.* 2012;94(3):966-972. doi:10.1016/j.athoracsur.2012.04.099

130.Treacy PJ, Barthe F, Bentellis I, Falagario UG, Prudhomme T, Imbert de la Phalecque L, et al. Is night-time surgical procedure for renal graft at higher risk than during the day? A single center study cohort of 179 patients*. Immun Inflamm Dis*. 2022;10(2):225-234. doi:10.1002/iid3.566

131.Tu DH, Qu R, Wang Q, Fu X. After-hours esophagectomy may pose additional risk to patients with esophageal cancer. *J Thorac Dis*. 2021;13(2):1118-1129. doi:10.21037/jtd-20-3141

132. Tu Y, Ning Y, Li K, Pan Z, Xie J, Yang S, et al. After-hour elective total knee arthroplasty does not affect clinical outcomes but negatively affects alignment*. Arch Orthop Trauma Surg*. 2023;143:2129–2134. doi:10.1007/s00402-022-04490-6

133.Turrentine FE, Wang H, Young JS, Calland JF. What is the safety of nonemergent operative procedures performed at night? A study of 10,426 operations at an academic tertiary care hospital using the American College of Surgeons national surgical quality program improvement database. *J Trauma.* 2010;69(2):313-319. doi:10.1097/TA.0b013e3181e49291

134.Vimalesvaran S, Ayis S, Krasemann T. Balloon atrial septostomy performed "out-of-hours": effects on the outcome. *Cardiol Young.* 2013;23(1):61-67. doi:10.1017/S1047951112000364

135.Vinden C, Nash DM, Rangrej J, Shariff SZ, Dixon SN, Jain AK, et al. Complications of daytime elective laparoscopic cholecystectomies performed by surgeons who operated the night before. *JAMA*. 2013;310(17):1837-1841. doi:10.1001/jama.2013.280372

136.Wan Z, Wang X, Li Y, Wan R. Impact of surgeon work duration prior to distal pancreatectomy on perioperative outcomes: a propensity score matching analysis. *BMC Surg.* 2021;21(1):54. doi:10.1186/s12893-021-01062-0

137.Wang B, Yao Y, Wang X, Li H, Qian H, Jiang L, et al. The start of gastrectomy at different time-of-day influences postoperative outcomes. *Medicine (Baltimore)*. 2020;99(21):e20325. doi:10.1097/MD.0000000000020325

138.Wendling-Keim DS, Binder M, Dietz HG, Lehner M. Timing of osteosynthesis of fractures in children changes the outcome. *Eur J Trauma Emerg Surg.* 2022;48(5):3461-3470. doi:10.1007/s00068-020-01464-4

139.Wu JX, Nguyen AT, de Virgilio C, Plurad DS, Kaji AH, Nguyen V, et al. Can it wait until morning? A comparison of nighttime versus daytime cholecystectomy for acute cholecystitis. *Am J Surg*. 2014;208(6):911-8; discussion 917-8. doi:10.1016/j.amjsurg.2014.09.004

140.Yaghoubian A, Kaji AH, Putnam B, de Virgilio C. Trauma surgery performed by "sleep deprived" residents: are outcomes affected? *J Surg Educ.* 2010;67(6):449-451. doi:10.1016/j.jsurg.2010.09.003

141.Yang Z, Takahashi T, Gerull WD, Hamilton C, Subramanian MP, Liu J, et al. Impact of nighttime lung transplantation on outcomes and costs. *Ann Thorac Surg.* 2021;112(1):206-213. doi:10.1016/j.athoracsur.2020.07.060

142.Yount KW, Lau CL, Yarboro LT, Ghanta RK, Kron IL, Kern JA, et al. Late operating room start times impact mortality and cost for nonemergent cardiac surgery. *Ann Thorac Surg*. 2015;100(5):1653-1658; discussion 1658-9. doi:10.1016/j.athoracsur.2015.04.131

143.van Zaane B, van Klei WA, Buhre WF, Bauer P, Boerma EC, Hoeft A, et al. Nonelective surgery at night and in-hospital mortality: prospective observational data from the European Surgical Outcomes Study. *Eur J Anaesthesiol*. 2015;32(7):477-485. doi:10.1097/EJA.0000000000000256

144.Zafar SN, Libuit L, Hashmi ZG, Hughes K, Greene WR, Cornwell 3^rd^ EE, et al. The sleepy surgeon: does night-time surgery for trauma affect mortality outcomes? *Am J Surg.* 2015;209(4):633-639. doi:10.1016/j.amjsurg.2014.12.015

145. Amirian I, Andersen LT, Rosenberg J, Gögenur I. Laparoscopic skills and cognitive function are not affected in surgeons during a night shift. *J Surg Educ.* 2014;71(4):543-550. doi:10.1016/j.jsurg.2013.12.007

146.Bykanov AE, Pitskhelauri DI, Zagidullin TR, Grachev NS, Danilov GV, et al. Effects of exogenous factors on spatial accuracy in neurosurgery. *J Clin Neurosci.* 2021;88:135-141. doi:10.1016/j.jocn.2021.03.039

147.Cumpanas AA, Bardan R, Ferician O, Latcu SC, Lazar OF, Duta C. The impact of tiredness on virtual reality robotic surgery skils. *Wideochir Inne Tech Maloinwazyjne*. 2020;15(2):298-304. doi:10.5114/wiitm.2020.93201

148.Cumpanas AA, Ferician O, Laţcu S, Duţă C, Bardan R, Lazăr FO. Does sleep deprivation alter virtual reality-based robotic surgical skills. *Wideochir Inne Tech Maloinwazyjne*. 2020;15(1):97-105. doi:10.5114/wiitm.2019.90565

149. Eastridge BJ, Hamilton EC, O’Keefe GE, Rege RV, Valentine RJ, Jones DJ, et al. Effect of sleep deprivation on the performance of simulated laparoscopic surgical skill. *Am J Surg.* 2003;186(2):169-174. doi:10.1016/s0002-9610(03)00183-1

150.Ganju A, Kahol K, Lee P, Simonian N, Quinn SJ, Ferrara JJ, et al. The effect of call on neurosurgery residents’ skills: implications for policy regarding resident call periods. *J Neurosurg.* 2012;116(3):478-482. doi:10.3171/2011.9.JNS101406

151.Grantcharov TP, Bardram L, Funch-Jensen P, Rosenberg J. Laparoscopic performance after one night on call in a surgical department: prospective study. *BMJ*. 2001;323(7323):1222-1223. doi:10.1136/bmj.323.7323.1222

152.Hegar MV, Truitt MS, Mangram AJ, Dunn EL. Resident fatigue in 2010: where is the beef? *Am J Surg.* 2011;202(6):727-731; discussion 731-2. doi:10.1016/j.amjsurg.2011.07.004

153.Jakubowicz DM, Price EM, Glassman HJ, Gallagher AJG, Mandava N, Ralph WP, et al.

Effects of a twenty-four hour call period on resident performance during simulated endoscopic sinus surgery in an accreditation council for graduate medical education-compliant training program. *Laryngoscope.* 2005;115(1):143-146. doi:10.1097/01.mlg.0000150689.77764.ad

154. Kocher H, Warwick J, Al-Ghnaniem R, Patel A. Surgical dexterity after a 'night out on the town'. *ANZ J Surg.* 2006;76(3):110-112. doi:10.1111/j.1445-2197.2006.03664.x

155.Leff DR, Aggarwal R, Rana M, Nakhjavani B, Purkayastha S, Khullar V, et al. Laparoscopic skills suffer on the first shift of sequential night shifts: program directors beware and residents prepare. *Ann Surg.* 2008;247(3):530-539. doi:10.1097/SLA.0b013e3181661a99

156.Leff DR, Orihuela-Espina F, Athanasiou T, Karimyan V, Elwell C, Wong J, et al. “Circadian cortical compensation”: a longitudinal study of brain function during technical and cognitive skills in acutely sleep-deprived surgical residents. *Ann Surg.* 2010;252(6):1082-1090. doi:10.1097/sla.0b013e3181ff449c

157.Taffinder NJ, McManus IC, Gul Y, Russel RC, Darzi A. Effect of sleep deprivation on surgeons' dexterity on laparoscopy simulator. *Lancet.* 1998;352(9135):1191. doi:10.1016/s0140-6736(98)00034-8

158. Tsafrir Z, Korianski J, Almog B, Many A, Wiesel O, Levin I. Effects of fatigue on residents’ performance in laparoscopy. *J Am Coll Surg.* 2015;221(2):564-570.e3. doi:10.1016/j.jamcollsurg.2015.02.024

159.Veddeng A, Husby T, Engelsen IB, Kent A, Flaatten H. Impact of night shifts on laparoscopic skills and cognitive function among gynecologists. *Acta Obstet Gynecol Scand.* 2014;93(12):1255-1261. doi:10.1111/aogs.12496

160.Waqar S, Park J, Kersey TL, Modi N, Ong C, Sleep TJ. Assessment of fatigue in intraocular surgery: analysis using a virtual reality simulator. *Graefes Arch Clin Exp Ophthalmol.* 2011;249(1):77-81. doi:10.1007/s00417-010-1531-6

161.Whelehan DF, Alexander M, Connelly TM, McEvoy C, Ridgway PF. Sleepy surgeons: a multi-method assessment of sleep deprivation and performance in surgery. *J Surg Res.* 2021;268:145-157. doi:10.1016/j.jss.2021.06.047

162.Yi WS, Hafiz S, Sava JA. Effects of night-float and 24-h call on resident psychomotor performance. *J Surg Res.* 2013;184(1):49-53. doi: 10.1016/j.jss.2013.03.029
